# Wide Distribution and Specific Resistance Pattern to Third-Generation Cephalosporins of *Enterobacter cloacae* Complex Members in Humans and in the Environment in Guadeloupe (French West Indies)

**DOI:** 10.3389/fmicb.2021.628058

**Published:** 2021-06-25

**Authors:** Matthieu Pot, Yann Reynaud, David Couvin, Célia Ducat, Séverine Ferdinand, François Gravey, Gaëlle Gruel, François Guérin, Edith Malpote, Sébastien Breurec, Antoine Talarmin, Stéphanie Guyomard-Rabenirina

**Affiliations:** ^1^Transmission, Reservoir and Diversity of Pathogens Unit, Pasteur Institute of Guadeloupe, Les Abymes, France; ^2^GRAM 2.0, Normandie University, UNICAEN, UNIROUEN, Caen, France; ^3^Department of Clinical Microbiology, Caen University Hospital, Caen, France; ^4^Laboratory of Clinical Microbiology, University Hospital of Guadeloupe, Pointe-à-Pitre/Les Abymes, France; ^5^Faculty of Medicine Hyacinthe Bastaraud, University of the Antilles, Pointe-à-Pitre, France; ^6^Centre for Clinical Investigation 1424, INSERM, Pointe-à-Pitre/Les Abymes, France

**Keywords:** *Anolis marmoratus*, cephalosporinase overproduction, *Enterobacter cloacae* complex, ESBL, *hsp60*, one health, phylogeny, Caribbean

## Abstract

Species belonging to *Enterobacter cloacae* complex have been isolated in numerous environments and samples of various origins. They are also involved in opportunistic infections in plants, animals, and humans. Previous prospection in Guadeloupe (French West Indies) indicated a high frequency of *E. cloacae* complex strains resistant to third-generation cephalosporins (3GCs) in a local lizard population (*Anolis marmoratus*), but knowledge of the distribution and resistance of these strains in humans and the environment is limited. The aim of this study was to compare the distribution and antibiotic susceptibility pattern of *E. cloacae* complex members from different sources in a “one health” approach and to find possible explanations for the high level of resistance in non-human samples. *E. cloacae* complex strains were collected between January 2017 and the end of 2018 from anoles, farm animals, local fresh produce, water, and clinical human samples. Isolates were characterized by the heat-shock protein 60 gene-fragment typing method, and whole-genome sequencing was conducted on the most frequent clusters (i.e., C-VI and C-VIII). The prevalence of resistance to 3GCs was relatively high (56/346, 16.2%) in non-human samples. The associated resistance mechanism was related to an AmpC overproduction; however, in human samples, most of the resistant strains (40/62) produced an extended-spectrum beta-lactamase. No relation was found between resistance in isolates from wild anoles (35/168) and human activities. Specific core-genome phylogenetic analysis highlighted an important diversity in this bacterial population and no wide circulation among the different compartments. In our setting, the mutations responsible for resistance to 3GCs, especially in *ampD*, were diverse and not compartment specific. In conclusion, high levels of resistance in non-human *E. cloacae* complex isolates are probably due to environmental factors that favor the selection of these resistant strains, and this will be explored further.

## Introduction

Bacteria in the *Enterobacter cloacae* complex (ECC) are widely distributed in numerous terrestrial and aquatic environments, and also in air and space equipment (Singh et al., 2018; Davin-Regli et al., 2019; Uchida et al., 2020). ECC are found in the gut microbiota of animals, including reptiles, mammals, and humans, and some studies have reported that the complex is endophytic (Liu et al., 2012; Singh et al., 2013; Davin-Regli et al., 2019). This bacterial complex also includes phytopathogenic clones (Humann et al., 2011), infection-causing strains in wild fauna and domestic animals (Haenni et al., 2016; Goldberg et al., 2019), and opportunistic pathogens that are involved in a wide variety of human infections, especially those associated with health care (Garinet et al., 2018).

The denomination “complex” refers to different *Enterobacter* species and subspecies, which are difficult to discriminate clearly only with phenotypic approaches. The current classification of ECC members is based on DNA analysis and specifically on partial sequence comparisons of heat-shock protein 60-gene (*hsp60*) fragments (Davin-Regli et al., 2019). The population structure of this complex was initially divided into 12 genetic clusters (C-I to C-XII) and a loosely knit group (C-xiii; Hoffmann and Roggenkamp, 2003). More recently, use of whole-genome sequencing (WGS) revealed a total of 22 phylogenetic clades (A–V), further illustrating the complex taxonomy of this genus (Chavda et al., 2016; Sutton et al., 2018). In addition, some phylogenetic clades are associated with previous *hsp60* clusters, and a novel cluster was reported (Beyrouthy et al., 2018).

The distribution of the ECC clusters was analyzed in studies related to clinical isolates (Kremer and Hoffmann, 2012; Garinet et al., 2018). Strains belonging to C-III, -VI, and -VIII usually predominated and were found to carry various determinants of antibiotic resistance (Stock et al., 2001; Hoffmann et al., 2005; Kremer and Hoffmann, 2012; Peirano et al., 2018). These clusters appeared to be adapted to the hospital environment (Paauw et al., 2008), and geographic specificities were recently reported (Zhou et al., 2018). Few studies, however, have provided information about the distribution of ECC members in animals and other sources, which limits comparisons and impedes understanding of their global epidemiology (Hoffmann and Roggenkamp, 2003; Kämpfer et al., 2008; Haenni et al., 2016).

*Enterobacter cloacae* complex belongs to the ESKAPE group which referred to *Enterococcus faecium*, *Staphylococcus aureus*, *Klebsiella pneumoniae*, *Acinetobacter baumannii*, *Pseudomonas aeruginosa* and *Enterobacter* spp., and is recognized as a “priority pathogen” due to its clinical relevance and association with antibiotic resistance and virulence genes (Rice, 2008; World Health Organization, 2017). All members of this complex have similar susceptibility to antibiotics (Davin-Regli et al., 2019), except fosfomycin (Stock et al., 2001). They are intrinsically resistant to aminopenicillin, the combination amoxicillin–clavulanic acid, and the first two generations of cephalosporins because they express an inducible *ampC* cephalosporinase. Under selection pressure, derepressed mutants often emerge, which overproduce cephalosporinase, conferring a high level of resistance to third-generation cephalosporins (3GCs) and increasing the minimum inhibitory concentration required for the fourth generation, such as cefepime (Guérin et al., 2015; Kohlmann et al., 2019; Mizrahi et al., 2020). Since the emergence of extended-spectrum beta-lactamase (ESBL), plasmidic acquisition of ESBL determinants has become an important mechanism in 3GCs resistance (3GC-R), especially among clinical ECC strains, as for most *Enterobacteriaceae.* These bacteria are also capable of acquiring genes that encode for carbapenemases, further restricting therapeutic management (Peirano et al., 2018). ESBL acquisition or cephalosporinase overproduction (CoP) appear to be distributed differently inside *hsp60* clusters, as illustrated by a higher frequency of ESBL production in C-VI and -VIII isolates (Stock et al., 2001; Garinet et al., 2018). As in other high resource countries, community resistance of ECCs to 3GCs is rare in Guadeloupe because of lower selection pressure (Guyomard-Rabenirina, 2016).

Although these bacteria have been described in the environment, the susceptibility of environmental strains is not well understood. There is little information in Guadeloupe, as in other South American countries, on the distribution of ECC members or on the resistance to antibiotics of strains isolated in the environment and in clinical samples. Resistance of *Enterobacteriaceae* to 3GCs was rare in community-acquired urinary tract infections (4.0%), due mainly to ESBL production (Guyomard-Rabenirina, 2016), in contrast to the high frequency of ESBL in clinical ECC (19.7%) from the University Hospital of Guadeloupe (S. Breurec, personal communication). In the local environment, we observed a high prevalence of 3GC-R *Enterobacteriaceae* carriage in feces of *Anolis marmoratus* (89/234, 38.0%), a small endemic lizard, and ECC members were the most prevalent among isolated strains (57/115, 49.5%; Guyomard-Rabenirina, 2016).

We conducted this study to investigate the distribution of ECC *hsp60* clusters isolated from various sources in Guadeloupe and to characterize their antibiotic susceptibility patterns in a “one health” approach. Genomic analysis was conducted on the main clusters in clinical samples (C-VI and C-VIII) and in different biotopes to compare the strains and to investigate the high level of 3GC-R in non-human samples.

## Materials and Methods

### Collection From Human

Between January and December 2018, clinical ECC isolates obtained during routine bacteriological diagnostics were collected prospectively from patients admitted to the University Hospital of Guadeloupe, a 900-bed teaching hospital in Pointe-à-Pitre/Les Abymes. The date and nature of the sample, and the results of antibiotic susceptibility analysis were recorded anonymously. Isolates were considered to be hospital-acquired if there were collected from patients hospitalized for more than 48 h after admission. The others were notified as to be community-acquired. Human samples were taken in accordance with the requirements of the local ethics committee and did not interfere with laboratory organization (reference A5_19_12_05_TRAMID).

### Sampling for Animal and Environmental Isolates

Between January 2017 and December 2018, ECC strains were isolated from water catchment areas, local fruits and vegetables, and fresh fecal samples from *A. marmoratus* and farm animals sampled at different sites in Guadeloupe.

Overall, 168 free-living adult lizards were caught and sampled at 17 sampling sites throughout the island (Supplementary Table 1). All the procedures were approved by the regional environment, planning, and housing agency and by the Guadeloupe National Park. The project was also approved by the Committee for Ethics in animal experiments of the French West Indies and Guyana (reference 971-2016-12-20-001). Animals were cared for and used according to French decree No. 2013-118 of 1 February 2013 on the protection of animals, which meets European Union Directive 2010/63 on the protection of animals used for experimental and other scientific purposes (Guyomard-Rabenirina et al., 2020). To study a possible association between ECC carriage and the degree of human activity at the site at which wild individuals were caught, the sampling location and the type of environment (urban, coastline, and mountain forest) were recorded (Supplementary Table 1). Urban sites were considered to be associated with moderate to high human activity, and the coastline and mountain forest environments with limited human impact. Fresh fecal samples from 34 pigs and 28 beef cattle were collected at the only slaughterhouse in Guadeloupe, located in Le Moule. The municipality of origin of the sampled animal was recorded (Supplementary Table 1). No information was available on antibiotic treatment. A total of 76 samples of fresh, locally produced fruits and vegetables were collected aseptically at four local markets (Bergevin, Convenance, Gourdeliane, and Saint-Jules). The market and the farm of origin were recorded, as well as the type of fertilizer used (organic or chemical) on each identified farm (*n* = 27 in Supplementary Table 1). A total of 40 raw water samples, which corresponded to drinking water before treatment, were collected at 24 catchment points, in collaboration with the regional health agency and the hygiene laboratory of the Pasteur Institute of Guadeloupe. Most of these sampling points were located in Basse-Terre. All samples were transported rapidly to the laboratory, stored at 5 ± 3°C and analyzed within 4 h.

### ECC Isolation and Antibiotic Susceptibility Analysis

All non-human samples were enriched. Animal stools, fruits, and vegetables were mixed with buffered peptone water. For water samples, 100 mL of serially diluted samples were filtered through a 0.45-μm membrane filter (Millipore, Guyancourt, France), and the membranes were placed in 9 mL of buffered peptone water solution. The non-selective enrichment broth for all samples was incubated for 16–20 h at 37°C. Then, 100 μL were inoculated onto chromogenic agar (CCA, CHROMagar, Paris, France). In addition, to increase the chances of detection of 3GC-R strains in the bacterial population, the same medium supplemented with ceftriaxone at 4 mg/L was also inoculated. Plates were incubated for 16–20 h at 37°C. This antibiotic was selected as it is considered to be a weak inducer of CoP as other 3GCs (Mizrahi et al., 2020). A maximum of five presumptive ECC colonies from each plate were isolated and identified by matrix-assisted laser desorption/ionization time-of-flight mass spectrometry (Shimadzu Biotech, Kyoto, Japan) and associated software.

Susceptibility to ampicillin (10 μg), amikacin (30 μg), amoxicillin–clavulanic acid (20–10 μg), aztreonam (30 μg), cefepim (30 μg), cefotaxime (5 μg), cefoxitin (30 μg), ceftazidime (10 μg), ciprofloxacin (5 μg), ertapenem (10 μg), gentamicin (10 μg), nalidixic acid (30 μg), temocillin (30 μg), ticarcillin (75 μg), tigecycline (15 μg), and trimethoprim–sulfamethoxazole (1.25–23.75 μg) was determined for all ECC strains by the disk diffusion method on Mueller-Hinton agar (Bio-Rad, Marnes-la-Coquette, France). Isolates were classified as resistant, intermediate, or susceptible according to the 2018 guidelines of CA-SFM/EUCAST^1^. Isolates of intermediate susceptibility were grouped with resistant isolates for data analysis (Supplementary Table 2). Production of ESBL was detected with the double-disk synergy test, according to CA-SFM/EUCAST recommendations.

### DNA Extraction

Total bacterial DNA was initially extracted from pure cultures with the Qiagen QIAamp DNA minikit (Qiagen, Hilden, Germany), according to the manufacturer’s instructions.

### ESBL Resistance Gene Screening

Extended-spectrum beta-lactamase-encoding genes were screened by polymerase chain reaction (PCR). On the basis of previous epidemiological evidence for ECC in the community, PCR was performed only for *bla*_CTX–M_ group 1 (Dallenne et al., 2010; Guyomard-Rabenirina, 2016). Amplicons were sequenced at Eurofins (Eurofins Genomic SAS, Les Ullis, France). Resistance genes were identified from the ResFinder database (Zankari et al., 2012).

### ECC *hsp60* Typing

As ECC members cannot be differentiated reliably with classic identification methods, we conducted sequence analysis of the partial *hsp60* gene, as described previously on 313 local strains (Hoffmann and Roggenkamp, 2003). PCR products were sequenced at Eurofins, and DNA sequences and chromatograms were analyzed with ApE software^2^. Maximum likelihood phylogenetic reconstruction was performed with RAxML in 100 replications (Stamatakis, 2014). The tree was drawn with iTOL (Letunic and Bork, 2019) and rooted with *Klebsiella aerogenes hsp60* partial sequence (AB008141.1; Hoffmann and Roggenkamp, 2003). Accession numbers for previously identified *hps60* cluster sequences are listed in Supplementary Figure 1. To complete the analysis, *hsp60* partial sequences were extracted from the assembled genomes of ECC ST873 strains, characterized, and added as a new ECC cluster (C-XIV – Clade S; Beyrouthy et al., 2018). Similar initial bioinformatic analysis was conducted on a selection of different ECC strains used and identified by Chavda et al. (2016), and Sutton et al. (2018; Supplementary Figure 1). We also included *hsp60* partial sequences of recently named strains: *E. wuhouensis*, *E. quasihormaechei* (Wang et al., 2020), *E. huaxiensis*, *E. chuandaensis* (Wu et al., 2019), and *E. oligotrophicus* (formerly *E. oligotrophica*) (Akita et al., 2019). The collected strains were assigned to a cluster on the basis of the reference data set used and bootstrap values (Supplementary Figure 1). When multiple strains were found in the same sample, only one in each cluster or resistance phenotype profile against beta-lactam antibiotics was conserved for the analysis, to avoid duplicates.

### Core-Genome Phylogenetic Analyses

To better describe possible circulation of ECC lineages collected from animals, fresh food, raw water, and humans and to gain understanding of the emergence of 3GC-R ECC from non-human samples, WGS was conducted on randomly selected strains in the two most prevalent *hsp60* clusters: C-VI (*n* = 45) and C-VIII (*n* = 86). Their origins were: 47 from *Anolis*, 5 from domestic animals, 14 from fresh produce, 10 from raw water samples, and 55 from human isolates. WGS was performed at the “Plateforme de microbiologie mutualisée” of the Pasteur International Bioresources network (Institut Pasteur, Paris, France). The method and software used for sequencing, quality checking and core-genome extraction were described previously (Guyomard-Rabenirina et al., 2020). Raw reads were trimmed and filtered with AlienTrimmer (Criscuolo and Brisse, 2014). Genomes were assembled with SPAdes software (Bankevich et al., 2012), and final quality was appreciated with QUAST and BUSCO score (Gurevich et al., 2013; Simão et al., 2015).

Total core single nucleotide polymorphisms (SNPs) were detected with Snippy software version 4.4.5, with GENC200 strain as reference for C-VI isolates and GENC071 strain for C-VIII^3^. Recombination elements were removed from the global core genome alignment with ClonalFrameML software (Didelot and Wilson, 2015), The two maximum likelihood phylogenetic reconstructions were performed with RAxML software in the GTR-CAT model and 1000 bootstrap replicates, and the trees were drawn with iTOL. Multilocus sequence typing was performed *in silico* with mlst software^4^ against the PubMLST database (Jolley et al., 2018), and virulence gene factors were identified with Abricate software^5^ associated with the Virulence Factor Database with a threshold of 95% coverage and 75% nucleotide identity (Chen et al., 2016). Abricate was used with these parameters to assess plasmid replicon and antibiotic resistance gene content associated with PlasmidFinder and ResFinder databases, respectively (Supplementary Table 3; Zankari et al., 2012; Carattoli et al., 2014).

### Focus on Cephalosporinase Genes and Mutations

In the ECC collection, 11 wild-type (WT)/CoP pairs from the same sample were analyzed for mutations. Each strain pair differed by fewer than 45 SNPs (Supplementary Table 4). Nine pairs belonging to ECC C-VIII and two to C-VI were selected. Most of the strains (14/22) were isolated from reptiles. Mutation analyses were performed with Snippy software, and the corresponding GenBank flat file format for each gene was retrieved from the NCBI website^6^ (Benson et al., 2018). The accession numbers of the sequences used as references were: NZ_CP012165.1 (*E. hormaechei* subsp. *oharae* strain 34978, complete genome) and NC_014121.1 (*E. cloacae* subsp. *cloacae* ATCC 13047 chromosome, complete genome). All genes which could be involved in CoP and 3GC-R phenotype were analyzed and listed in Supplementary Table 4 (see **Reference.gbk**). An in-house Perl script was used to extract results from files generated by Snippy and to create a table of the numbers of non-synonymous and synonymous gene mutations in the ECC sequences retrieved from the same host but with different antibiotic resistance profiles.

### Statistical Analyses

The analyses and data collection were performed with Microsoft Access 2003. Pearson’s χ^2^ or Fisher’s exact test was used. *P* values < 0.05 were considered significant.

## Results

Between January 2017 and December 2018, 313 unique ECC strains were isolated from various sample types, after removal of duplicates (Table 1). In non-human isolates, ECC strains (WT and 3GC-R) were isolated from 57.9% (44/76) of fresh produce and 42.9% (72/168) of anole samples; half of the raw water samples (20/40) were positive, while ECC strains were isolated from only 29.0% of livestock samples (18/62). Overall, resistance to 3GCs was found in 16.2% of non-human samples (56/346) and was due only to CoP. Most of these 3GC-R strains exhibited a 6 mm inhibition diameter on Mueller-Hinton agar plate for cefotaxime and ceftazidime antibiotics (data not shown). The prevalence of CoP was higher in anole samples (35/168, 20.8%) than in vegetable (13/76, 17.1%), livestock (6/62, 9.7%), or water isolates (2/40, 5.0%). Most of the anoles were trapped in areas impacted by human activities (*n* = 112, 66.7%); however, no significant difference was found in the rates of CoP by degree of human activity (Table 2). A positive association was found between the prevalence of positive for ECC and use of organic fertilizer (11/11) rather than chemical fertilizer (4/12; *P* = 0.001) from the 27 identified farms, but a similar association was not found for the CoP rate (*P* = 0.15, i.e., 4/11 and 1/12; Supplementary Table 1).

**TABLE 1:** Prevalence of resistance to each tested antibiotic of *E. cloacae* complex (ECC) isolates.

	Human (*N* = 107)				*Anolis* (*N* = 110)		Fresh produce (*N* = 52)		Water (*N* = 23)		Livestock (*N* = 21)	
					
Antibiotic	WT	CoP	ESBL	CP	WT	CoP	WT	CoP	WT	CoP	WT	CoP
	(*N* = 45)	(*N* = 21)	(*N* = 40)	(*N* = 1)	(*N* = 70)	(*N* = 40)	(*N* = 38)	(*N* = 14)	(*N* = 21)	(*N* = 2)	(*N* = 15)	(*N* = 6)
Ticarcillin	3 (6.7)	21 (100.0)	40 (100.0)	1 (100.0)	4 (5.7)	36 (90.0)	4 (10.5)	14 (100.0)	–	2 (100.0)	1 (6.7)	6 (100.0)
Temocillin	–	17 (81.0)	20 (50.0)	1 (100.0)	1 (1.4)	17 (42.5)	–	9 (64.3)	–	–	–	2 (33.3)
Cefotaxime	–	21 (100.0)	40 (100.0)	1 (100.0)	–	33 (82.5)	–	14 (100.0)	–	2 (100.0)	–	6 (100.0)
Ceftazidime	–	19 (90.5)	40 (100.0)	1 (100.0)	–	40 (100.0)	–	14 (100.0)	–	2 (100.0)	–	6 (100.0)
Aztreonam	–	14 (66.7)	39 (97.5)	1 (100.0)	–	34 (85.0)	–	13 (92.9)	–	1 (50.0)	–	6 (100.0)
Cefepim	–	–	40 (100.0)	1 (100.0)	–	–	–	–	–	–	–	–
Ertapenem	–	1 (4.8)	1 (2.5)	1 (100.0)	–	20 (50.0)	–	9 (64.3)	–	1 (50.0)	–	4 (66.7)
Nalidixic acid	4 (8.9)	4 (19.0)	38 (95.0)	1 (100.0)	–	–	–	–	1 (4.8)	–	–	–
Ciprofloxacin	3 (6.7)	4 (19.0)	36 (90.0)	1 (100.0)	–	–	–	–	–	–	–	–
Gentamicin	–	2 (9.5)	31 (77.5)	–	–	–	–	–	–	–	–	–
Amikacin	–	–	1 (2.5)	–	–	–	–	–	–	–	–	–
Tigecycline	3 (6.7)	1 (4.8)	5 (12.5)	–	–	–	–	–	–	–	1 (6.7)	–
Trimethoprim – sulfamethoxazole	3 (6.7)	2 (9.5)	35 (87.5)	1 (100.0)	–	–	–	–	–	–	1 (6.7)	–

*ECC strains (*n* = 313) were grouped according to their origin and resistance to beta-lactam antibiotics: wild-type (WT), cephalosporinase overproduction (CoP), extended-spectrum beta-lactamase (ESBL), or carbapenemase production (CP). All strains were resistant to ampicillin, amoxicillin–clavulanic acid, and cefoxitin (i.e., “R” Supplementary Table 2).*

**TABLE 2:** Carriage of *E. cloacae* complex and third-generation cephalosporin-resistant (3GC-R) strains in the *Anolis* population, according to degree of human activity.

.Sampling site			*Anolis marmoratus* sampled	*E. cloacae* complex isolation			
		
Type	(*N* = 17)^a^	Degree of human activity	(*N* = 168)	Total of positive sample	*p-value*	3GC-R	*p-value*
				*N* (%)		*N* (%)	
Urban	11	Moderate-high	112	53 (47.3)	0.1	25 (22.3)	0.5
Coastline or mountain forest	6	Limited	56	19 (33.9)		10 (17.9)	

*^*a*^GPS coordinates are provided in Supplementary Table 1.*

Most of human isolates were hospital-acquired (95/107) and 3GC-R were mainly associated to ESBL production (40/62; Table 1 and Supplementary Table 2). Genes encoding for ESBL were identified in all clinical strains positive in the double-disk synergy test (40/107, 37.4%). Amplicon sequencing revealed a *bla*_CTX–M–15_ gene in 38 isolates (95.0%). In addition, WGS allowed us to identify a *bla*_GES–7_ gene on the two last ESBL producers (GENC084, GENC220). One strain of human origin carried a *bla*_OXA–48_ gene (GENC133).

Co-resistance against beta-lactams (including 3GCs) and other antibiotic families was observed mainly in human associated strains and was usually to fluoroquinolones (41/62, 66.2%; Table 1). Co-resistance to gentamicin and trimethoprim–sulfamethoxazole was also found frequently, in 29 3GC-R clinical strains (46.8%) but not in samples from other sources. Only one strain from a raw water sample was resistant to nalidixic acid (ECC403), and one strain from livestock was notified resistant to trimethoprim–sulfamethoxazole and tigecycline (ECC408).

### Distribution of *hsp60* Clusters

Strain diversity was first investigated with *hsp60* typing. A neighbor-joining tree was constructed with 129 alignment patterns of 313 partial *hsp60* sequences from the different isolates, comprising 131 strains from the animal collection (110 from anole, 9 from pig, and 12 from beef cattle), 23 from water, 52 from fresh produce, and 107 from clinical samples (Table 3 and Supplementary Figure 1). All *Enterobacter* Hoffman clusters except C-VII (*E. hormaechei* subsp. *hormaechei*) were represented in our study, including C-XIV (*n* = 2) in human and domestic animal samples (Hoffmann and Roggenkamp, 2003; Beyrouthy et al., 2018); 28 strains, mainly human isolates (14/28), did not correspond to any of the 14 previously defined *hsp60* clusters. They were grouped into six undefined *hsp60* clusters (recorded as UD1–6), which included ECC clades recently identified by WGS (K,L,P,N,T) and *E. oligotrophica* (Table 3, Supplementary Table 2, and Supplementary Figure 1; Chavda et al., 2016; Sutton et al., 2018; Akita et al., 2019).

**TABLE 3:** Distribution of *E. cloacae* complex (ECC) members in samples of different origin.

*Enterobacter* species or subspecies names	*hsp60* cluster^a^	WGS clade^a^	Number of collected strains	Origin N (%)					3GC-R *N* (%)		ESBL^c^*N* (%)
					
				Human	*Anolis*	Fresh produce	Water	Livestock	Human	Non-human^d^	
			(*N* = 313)	(*N* = 107)	(*N* = 110)	(*N* = 52)	(*N* = 23)	(*N* = 21)	(*N* = 62)	(*N* = 62)	(*N* = 40)
*E. asburiae*	I	J	13	6 (46.1)	–	2 (15.4)	4 (30.8)	1 (7.7)	4 (30.8)	–	3 (23.1)
*E. kobei*	II	Q	3	2 (66.7)	–	1 (33.3)	–	–	–	–	–
*E. hormaechei* subsp. *hoffmannii*	III	D	1	1 (100.0)	–	–	–	–	1 (100.0)	–	–
*E. roggenkampii*	IV	M	18	4 (22.2)	6 (33.3)	6 (33.3)	1 (5.6)	1 (5.6)	3 (16.7)	3 (16.7)	–
*E. ludwigii*	V	I	1	1 (100.0)	–	–	–	–	–	–	–
*E. hormaechei* subsp. *xiangfangensis*	VI	A	48	28 (58.3)	6 (12.5)	9 (18.8)	–	5 (10.4)	22 (45,8)	10 (20.8)	16 (33.3)
*E. hormaechei* subsp. *oharae*	VI	C	3	1 (33.3)	–	2 (66.7)	–	–	–	1 (33.3)	–
*E. hormaechei* subsp. *hormaechei*	VII	E	–	–	–	–	–	–	–	–	–
*E. hormaechei* subsp. *steigerwaltii*	VIII	B	87	31 (35.6)	43 (49.4)	3 (3.5)	10 (11.5)	–	13 (14,9)	18 (20,7)	7 (8.0)
*E. bugandensis*	IX	R	32	10 (31.2)	7 (21.9)	9 (28.1)	2 (6.3)	4 (12.5)	4 (12.5)	6 (18.6)	1 (3.1)
*E. cloacae* subsp. *cloacae*	XI	G	19	5 (26.3)	1 (5.3)	5 (26.3)	1 (5.3)	7 (36.8)	2 (10.5)	1 (5.3)	1 (5.3)
*E. cloacae* subsp. *dissolvens*	XII	H	21	2 (9.5)	8 (38.1)	8 (38.1)	1 (4.8)	2 (9.5)	1 (4,8)	4 (19,0)	–
*E. cloacae* complex	xiii	na	37	1 (2.7)	31 (83.8)	3 (8.1)	2 (5.4)	–	–	16 (43.2)	–
*E. quasihormaechei*	XIV	S	2	1 (50.0)	–	–	–	1 (50.0)	1 (50.0)	–	1 (50.0)

Undefined clusters^b^	UD1	P	1	1 (100.0)	–	–	–	–	–	–	–
	UD2	N	2	–	1 (50.0)	1 (50.0)	–	–	–	1 (50.0)	–
	UD3	K	2	–	1 (50.0)	1 (50.0)	–	–	–	–	–
	UD4	L	14	13 (92.9)	–	–	1 (7.1)	–	11 (78.6)	1 (7.1)	11 (78.6)
	UD5	na	1	–	1 (100.0)	–	–	–	–	–	–
	UD6	T	8	–	5 (62.5)	2 (25.0)	1 (12.5)	–	–	1 (12.5)	–

*^*a*^ Details of the strains and sequences used as reference are provided in Supplementary Figure 1 and in Supplementary Table 2.^*b*^ Undefined cluster numbers proposed in this article.^*c*^ Extended-spectrum beta-lactamase (ESBL) producers were only recovered from human samples. ^*d*^ Most of the third-generation cephalosporin-resistant (3GC-R) strains isolated from non-human samples were identified from CCA medium supplemented with ceftriaxone 4 mg/L (56/62; see Supplementary Table 2); na, not attributed.*

Cluster VIII was best represented, except in livestock and fresh produce. C-VI was the second most frequent in clinical strains, whereas C-xiii was over-represented among anole isolates (28.2%), and *E. asburiae* was more frequent in water samples (C-I, 17.4%). In livestock, C-XI, -VI, and -IX predominated (Table 3 and Supplementary Figure 1). CoP strains were found in nearly all clusters except C-II, -V, and UD1, 3 and 5. The highest rate of CoP strains was found in cluster VIII (Table 3).

### Genetic Analysis of ECC Clusters VI and VIII Populations

**FIGURE 1:**
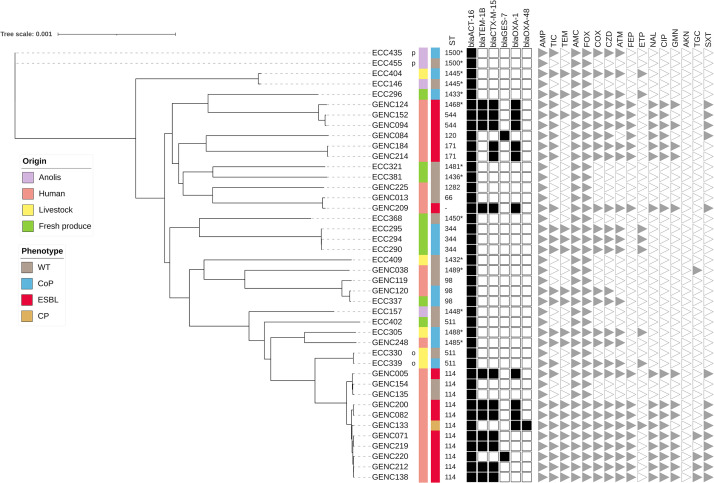
Maximum likelihood phylogenetic tree of *E. cloacae* complex C-VI – clade A isolates recovered in Guadeloupe (*n* = 42). Maximum likelihood phylogenetic reconstructions were performed with RAxML software (1000 bootstrap replicates), and the tree was drawn with iTOL. Hosts and phenotypes are indicated by vertical colored strips. The letters indicate specific wild-type and cephalosporinase overproduction pairs in the same sample. New sequence types (STs) identified in this study are indicated by a star, while unknown ST is denoted by a dash. Only genes that confer resistance to beta-lactam antibiotics were included. They were characterized by ResFinder and are indicated by black squares; all genetic details are provided in Supplementary Table 3. Antibiotic resistance profiles are indicated by gray triangles; AKN, amikacin; AMC, amoxicillin–clavulanic acid; AMP, ampicillin; ATM, aztreonam; CIP, ciprofloxacin; COX, cefotaxime; CZD, ceftazidime; ETP, ertapenem; FEP, cefepim; FOX, cefoxitin; GMN, gentamicin; NAL, nalidixic acid; TEM, temocillin; TGC, tigecycline; TIC, ticarcillin; and SXT, trimethoprim–sulfamethoxazole.

The WGS generated a mean of 149.87 bp paired-end reads, with an estimate coverage of 77.851-fold (AlienTrimmer; Criscuolo and Brisse, 2014). Quality of the assembly indicated a mean N50 of 309532 (minimum 43293, maximum 782933), and a mean single-copy BUSCO score of 98.6% completeness (Gurevich et al., 2013; Simão et al., 2015). Of the 45 sequenced isolates assigned to C-VI, 42 belonged to *E. hormaechei* subsp. *xiangfangensis* (clade A), and only three were identified as *E. hormaechei* subsp. *oharae* (clade C; Table 3; Sutton et al., 2018). These clade C strains were isolated from fresh produce and human samples (ECC 312, ECC336, and GENC003) and were not conserved for further analysis. Maximum likelihood phylogenetic analysis of *E. hormaechei* subsp. *xiangfangensis* strains indicated wide diversity among isolates (mean SNP of all isolates *n* = 17973, minimum *n* = 4, and maximum *n* = 22821). Fifteen strains were not assigned to a well-known sequence type (ST) or identified as new, whereas the others belonged to nine clearly identified sequence types (Figure 1). The largest cluster contained 11 strains in the international clone ST114 (mean SNP between isolates *n* = 935, minimum *n* = 4, and maximum *n* = 2122), which were isolated only from humans. Most of the strains (8/11) were ESBL producers. Some genetically related clusters with isolates of different origins were observed, comprising a cluster with three ST98 strains (two from humans and one from a cucumber; SNP mean = 484, minimum = 68) and a second with three vegetables strains belonging to ST344. The samples, taken at the same time at Bergevin market on 12 January 2018 (Supplementary Table 1), were from two farms, had the same antibiotic resistance profile (3GC-R), and differed by a mean of 45 SNPs (minimum = 36). One strain from *Anolis* clustered with an isolate from fresh produce, with only 65 SNPs difference (Figure 1).

**FIGURE 2:**
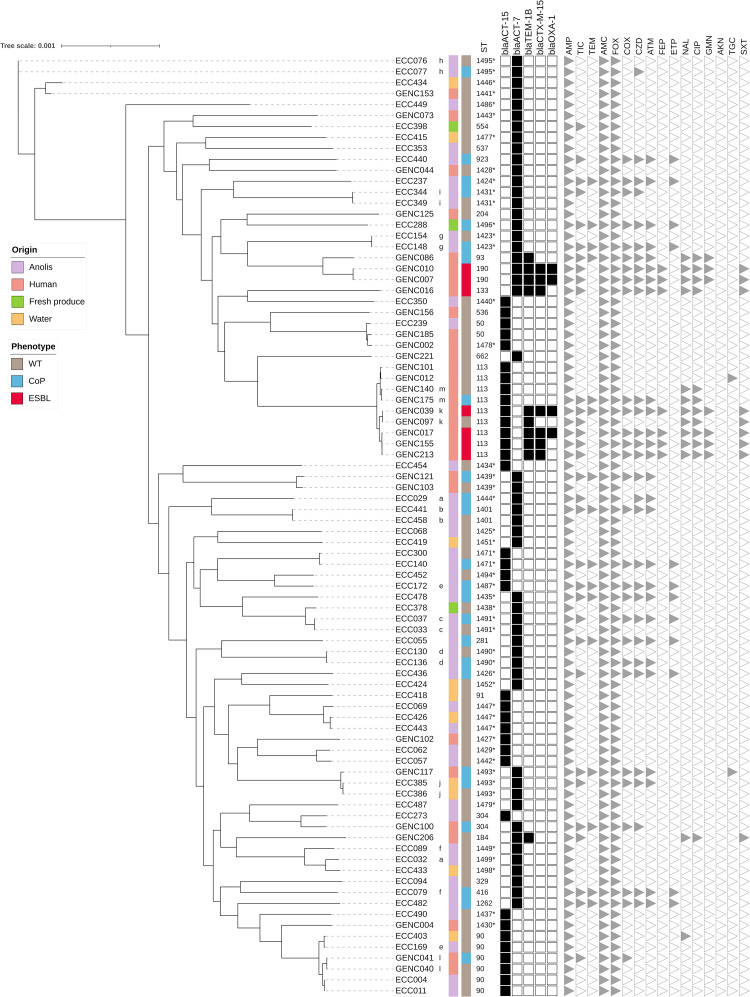
Maximum likelihood phylogenetic tree of *E. cloacae* complex C-VIII isolates recovered in Guadeloupe (*n* = 86). Maximum likelihood phylogenetic reconstructions were performed with RAxML software (1000 bootstrap replicates), and the tree was drawn with iTOL. Hosts and phenotypes are indicated by vertical colored strips. The letters indicate specific wild-type and cephalosporinase overproduction pairs in the same sample. New sequence types (STs) identified in this study are indicated by a star. Only genes that confer resistance to beta-lactam antibiotics were included. They were characterized by ResFinder and are indicated by black squares; all genetic details are provided in Supplementary Table 3. Antibiotic resistance profiles are indicated by gray triangles; AKN, amikacin; AMC, amoxicillin–clavulanic acid; AMP, ampicillin; ATM, aztreonam; CIP, ciprofloxacin; COX, cefotaxime; CZD, ceftazidime; ETP, ertapenem; FEP, cefepim; FOX, cefoxitin; GMN, gentamicin; NAL, nalidixic acid; TEM, temocillin; TGC, tigecycline; TIC, ticarcillin; and SXT, trimethoprim–sulfamethoxazole.

Phylogenetic analysis of C-VIII revealed greater diversity, especially among anole strains (mean SNP for all isolates *n* = 23125, minimum *n* = 8, and maximum *n* = 27680). Various lineages were observed in the same sample (samples a, e, and f in Figure 2 and Supplementary Table 4). *In silico* analyses revealed the presence of two main groups in this second tree. The first (C-VIII-A, *n* = 32) consisted mainly of clinical isolates (20/32; mean SNP among isolates *n* = 26166, minimum *n* = 8, and maximum *n* = 31449), while reptile strains predominated in the second one (C-VIII-B, 31/49). ESBL producers were found only in C-VIII-A and, as expected, only in human isolates. Like ST114 in ECC C-VI, ST113 was well represented in human samples for C-VIII (*n* = 9), and four were ESBL producers. This ST was not found in other biotopes (Supplementary Table 3).

As observed in the C-VI phylogenetic tree, some strains of different origins were genetically related. ST90 was recovered from three different *A. marmoratus*, one water sample, and one human (two strains from the same patient, l in Figure 2 and Supplementary Table 4; mean SNP among isolates *n* = 1283, minimum *n* = 8, and maximum *n* = 2221). Most of these strains presented a CoP (5/6). Two other STs found in wild fauna and human samples clustered together: ST50, (ECC239–GENC185, 225 SNPs) and ST304 (ECC273–GENC100, 7698 SNPs). The human strain GENC117 shared the same target sequence of seven housekeeping genes with ECC386 isolated from water and a difference of 123 SNPs (i.e., new ST1493). One strain from *Anolis* clustered with an isolate from water, with a difference of only 101 SNPs (ECC443–ECC426). Two strains isolated from two *Anolis* 28 km apart clustered, with a difference of only 81 SNPs (ECC140–ECC300).

Few different resistance gene types were shared by ECC isolates from the 5 origins (Supplementary Table 3). The genes belonged to *bla*_ACT_, and one gene conferred resistance to fosfomycin (*fosA*). Genes encoding for efflux pumps (*oqxA*–*oqxB* and *mdfA*) were observed in human and non-human isolates. Genes encoding for cephalosporinase were specific for each cluster. *E. hormaechei* subsp. *xiangfangensis* was related to the *bla*_ACT–16_ gene type, while *bla*_ACT–15_ and *bla*_ACT–7_ were associated with *E. hormaechei* subsp. *steigerwaltii*. Human strains belonging to C-VI and C-VIII expressed more resistance genes (mean, nine) than those from other compartments (mean, three; Supplementary Table 3). All sequenced and analyzed strains resistant to fluoroquinolones (28/128; except GENC200) harbored quinolone resistance genes (*qnrB1*, *qnrB19* or *qnrS2*). Regarding the plasmid distribution, 50.0% (64/128) of the sequenced C-VI and C-VIII carried at least one identifiable replicon type. Among them Col (48/64), IncFII (*n* = 24), IncHI2 (*n* = 21), and IncFIB (*n* = 20) were the most often identified. Only 24.3% (18/74) of non-human strains presented at least one replicon. This prevalence was higher in the human collection (46/54, 85.2%), and whole-genome sequenced ESBL-producing ECC were especially associated with an IncHI2 signature (19/22, 86.4%; Supplementary Table 3). We also identified genes associated with virulence in C-VI and C-VIII populations in both human and non-human strains, which are involved in bacterial adherence, iron uptake, motility, or toxin production (Supplementary Table 3). Globally, human and non-human isolates expressed a similar number of virulence genes (mean twenty-one). All sequenced C-VIII strains (*n* = 86) harbored genes involved in the salmochelin siderophore system (*iroB*, *iroC*, *iroD*, *iroE*, and *iroN*), while this system was not found in the C-VI population. The yersiniabactin system (*fyuA, irp1, irp2, ybtA, ybtE, ybtP, ybtQ, ybtS, ybtT, ybtU*, and *ybtX*) was identified in two non-human strains belonging to ST90 (ECC169 and ECC403).

### Cephalosporinase Genes Mutation Profile Analysis

In isolates analyzed for cephalosporinase mutation, alignment of the AmpD protein sequences belonging to the WT ECC showed relatively good conservation (with an overall identity of 94.65%). Few mismatches were found (Supplementary Table 4). Comparison of 3GC-R with WT isolates from the same sample indicated that differences in mutations were found mainly in the *ampD* gene. Significant mutations are listed in the last column of Supplementary Table 4. Furthermore, other genes such as *dacB* and *ramR* could play a non-negligible role in the differentiation of resistant and susceptible strains. Our analysis indicates non-synonymous mutations different from those was observed previously, although Ala60Val substitution in *ampD* was also detected in ECC033 and ECC037 (Flury et al., 2016).

## Discussion

This study of ECC diversity in samples from different compartments in Guadeloupe showed by *hsp60* typing analysis that six clusters (C-IV, -VI, -VIII, -IX, -XI, and -XII) are identified in more than two thirds of the strains (228/313, 72.8%), and most are present in all sample types. Although this typing method is limited and focus on only one gene (Hoffmann and Roggenkamp, 2003; Wu et al., 2020), it indicates a high degree of diversity of this bacterial complex which is present in a wide variety of compartments.

The *E. hormaechei* metacluster predominated especially with C-VI (51/313, 16.3%) and C-VIII (87/313, 27.8%), which were also the most frequent clusters in human infections (27.1 and 29.0%, respectively). Surprisingly, C-III was rare in Guadeloupe, with only one strain in a human sample, whereas this *E. hormaechei* subspecies is one of the most frequently reported in clinical studies (Hoffmann and Roggenkamp, 2003; Kremer and Hoffmann, 2012; Garinet et al., 2018). In contrast, we notified the high prevalence of the *hsp60* UD4 cluster in our set of clinical isolates which should referred to ECC clade L (Sutton et al., 2018), and a possible new successful ESBL-producing lineage.

Among other important clusters, *E. bugandensis* (C-IX) accounted for 10 of the 107 clinical strains, and 22 isolates were recovered from non-clinical samples. This species was found mainly in fresh produce (9/22) and reptile feces (7/22). It has been reported in wild fauna, livestock, and the environment in only a few studies (Khanna et al., 2013; Singh et al., 2018; Matteoli et al., 2019; Wu et al., 2020). *E. bugandensis* has been described as the most virulent and pathogenic of all ECC strains (Doijad et al., 2016; Pati et al., 2018), which raises concern, as fresh produce may be the origin of dissemination of virulent pathogens in the community, as for some *Escherichia coli* lineages (Luna-Guevara et al., 2019).

As C-VI and -VIII were the most prevalent *hsp60* clusters in this collection, the WGS analysis was conducted to investigate their potential clone diffusion among human, animal, and environmental samples. Among C-VI, *E. hormaechei* subsp. *xiangfangensis* was overrepresented in comparison to the other related subspecies *E. hormaechei* subsp. *oharae* (3/45) as reported in previous studies (Peirano et al., 2018; Sutton et al., 2018). Core-genome analysis showed that clonal spread at hospital level was limited to a few genetic backgrounds which were previously found in human infections (i.e., ST113 and ST114; Peirano et al., 2018; Siebor et al., 2019). In accordance with our observations, they have rarely been observed in samples of other origins, except from companion and wild animals (Haenni et al., 2016; Harada et al., 2017; Goldberg et al., 2019). Different sample types shared a few lineages, one being ST90 (*n* = 5), which is also involved in human and animals infections (Peirano et al., 2018; Zhu et al., 2020). Moreover, two ST90 strains isolated from an anole and a raw water sample harbored genes encoding for the yersiniabactin system. The presence of this highly pathogenic island has previously been described in other *Enterobacteriaceae* species and was associated with a specific *E. hormaechei* clone that caused a hospital outbreak in Netherlands (Paauw et al., 2009). Another ST previously found in a clinical sample (ST98) was present in both a human collection and fresh produce (Izdebski et al., 2015). ST344 clones were found in three fresh products but from two different market stands, suggesting manual strain circulation.

This study also showed a high frequency of 3GC-R ECC members in non-human samples such as livestock and fresh local produce, and confirmed previous observations on the local anole population (Guyomard-Rabenirina, 2016). Three hypotheses have been proposed to explain this high prevalence of CoP ECC strains in these compartments. The first one was a human origin of these resistant strains, with the spread of successful lineages among compartments; however, *hsp*60 clusters distribution within each sample type and the WGS analysis of C-VI and -VIII, indicated a wide diversity, which was not in favor of exchanges. Although some whole-genomes sequenced strains isolated from humans, lizards, and other origins were genetically close, most of them were grouped separately.

The second hypothesis was an impact of human activities, which could exert selective pressure for resistant ECC strains, as few international ST were found in non-clinical isolates (ST90, ST98; Izdebski et al., 2015; Peirano et al., 2018). No significant difference was found between *Anolis* individuals sampled near or far from areas of human activity, which is congruent with the results of a study on 3GC-R *E. coli* carriage in lizard in Guadeloupe (Guyomard-Rabenirina et al., 2020). To go further in a recent survey, *Anolis* (*n* = 20) and other wild animals (*n* = 67; rat, bird, toad, and cockroach) living near the hospital sewers and at the associated wastewater treatment plant were sampled. Among them, 21.8% (19/87) carried 3GC-R ECC including one *Anolis*. These strains were exclusively ESBL-producers, and WGS analysis indicated the presence of a ST114 lineage closely related to human samples and the dissemination of an IncHI2/*bla*_CTX–M–15_ plasmid. Taken together, these results suggested that only specific polluted environments associated with an important selective pressure are in favor of a large dissemination and maintenance of human related resistant strains in the wild fauna compartment (Pot et al., 2021). This second hypothesis is less well supported for fruits and vegetables, as the origin of the resistant clones in such samples could be multifactorial (Hölzel et al., 2018). Overall, the prevalence of 3GC-R clones in fresh produce was higher than in a previous larger collection, but only for CoP clones (van Hoek et al., 2015), in contrast to other published reports of ESBL producers (van Hoek et al., 2015; Hölzel et al., 2018). Our results suggest that organic fertilization is associated with a higher load of ECC strains in fresh produce but is not correlated with higher counts of CoP ECC, as described previously (Marti et al., 2013).

As it has been suggested that ECC members have higher mutation rates due to derepression than other genera with constitutive *ampC* (Kohlmann et al., 2018), we explored a third hypothesis, that non-human strains have a greater ability to acquire specific mutations in genes encoding for AmpC. This hypothesis was rapidly excluded by analysis of 11 WT/CoP pairs, in which most mutations were shared by strains from the different compartments and especially mutations in *ampD*, which has been suggested to be the leading mechanism in ECC with CoP in human samples (Guérin et al., 2015).

Our results increase the understanding of reservoirs definition and sources of ECC infections in a tropical setting. Several limitations should be pointed in this study. First, human isolates were recovered from only infectious sites and were mostly specified to be hospital-acquired. Nevertheless, since ECC members are opportunistic pathogens and belong to *Enterobacteriaceae*, most of infections came from endogenous colonizing strains (Gorrie et al., 2018). As we used selective medium to facilitate resistance detection, it could led to the overrepresentation of 3GC-R ECC in non-human isolates. This selection bias was limited by using ceftriaxone as selective antibiotic, described as a weak CoP inducer (Mizrahi et al., 2020). Moreover, previous authors indicated a relatively low rate of derepressed mutant in ECC population (3 × 10^–8^; Kohlmann et al., 2018), although we did not estimate the mutation rate in our non-human samples due to the presence of various cultivable genus and species in agar plates.

## Conclusion

Our findings highlight the widely diverse distribution of ECC members in non-human and human samples. We found a high prevalence of 3GC-R ECC in non-human samples, due exclusively to CoP. None of our hypotheses could explain this prevalence, and higher mutation rate is not excluded. These results suggest that this characteristic confers a selective advantage for these strains. Unknown persistent environmental factors, which should be further explored, may favor such overproduction.

## Data Availability Statement

The 313 partial *hsp60* sequences are available in Supplementary Table 2 with strain details, while the whole-genome sequences of C-VI, clade A (*n* = 42) and C-VIII (*n* = 86) are deposited in GenBank under BioSample accession numbers SAMN15680734 to SAMN15680861 (Supplementary Table 3).

## Ethics Statement

The parts of this project involving human participants were reviewed and approved by Commission de recherche éthique, Direction de la recherche et de l’innovation, CHU de la Guadeloupe (A5_19_12_05_TRAMID). Written informed consent for participation was not required for this study in accordance with the national legislation and the institutional requirements. The animal study was reviewed and approved by the Committee for Ethics in animal experiments of the French West Indies and Guyana (reference 971-2016-12-20-001). Written informed consent for participation was not obtained from the owners because feces were collected from livestock animals at the slaughterhouse, just before slaughter. No data concerning farm was avalaible. Only the municipality origin of screened animals batchs was recorded.

## Author Contributions

MP, SG-R, and AT conceived and designed the study. MP, SB, CD, SF, GG, SG-R, EM, and AT collected biological samples, isolates, and epidemiological data. MP, DC, FGu, SG-R, FGr, AT, and YR analyzed the data. MP, SB, DC, SG-R, and AT wrote the manuscript. All the authors critically revised and approved the final version of the manuscript.

## Conflict of Interest

The authors declare that the research was conducted in the absence of any commercial or financial relationships that could be construed as a potential conflict of interest.

## Footnotes

1 http://www.sfm-microbiologie.org

2 https://jorgensen.biology.utah.edu/wayned/ape/

3 https://github.com/tseemann/snippy

4 https://github.com/tseemann/mlst

5 https://github.com/tseemann/abricate

6 https://www.ncbi.nlm.nih.gov/genbank/

## Supplementary Material

Supplementary Figure 1 Maximum likelihood phylogenetic tree based on the partial sequence *hsp60* gene of *E. cloacae* complex (ECC) isolates recovered in Guadeloupe (*n* = 313). Maximum likelihood phylogenetic reconstructions were performed with RAxML software (100 bootstrap replicates), and the tree rooted with *Klebsiella aerogenes hsp60* sequence (AB008141.1) was drawn with iTOL. All the sequences used as reference in the tree have a corresponding GenBank accession number, and only sequences extracted from whole-genome of ECC strains were labeled with current names. Each *hsp60* cluster is identified by a specific color, and bootstrap values ≥ 60 are indicated. *Hsp60* sequences from C-X, which correspond to *Lelliottia nimipressuralis* (formerly *Enterobacter nimipressuralis*) are inserted (AJ567887.1, AJ567900.1). For better readability, C-IV nodes are grouped. The hosts of each local strain are specified by a circle (human), a square (livestock), a star (fresh produce), a right-pointing triangle (water), or a left-pointing triangle (*Anolis*). Phenotypes are indicated by a white form for wild-type against beta-lactam antibiotics, and a black form for third-generation cephalosporin-resistant strains. The global distribution is provided in Table 3, and all *hsp60* partial sequences are available in Supplementary Table 2. List of ECC partial *hps60* sequences included in this figure (associated cluster: C or UD, and accession number): **C-I**: AJ417140.1, AJ567846.1, AJ567893.1, FXLQ00000000; **C-II**: AJ567849.1, AJ567862.1, AJ567886.1, AJ567888.1, AJ567899.1, CP017181.1, **C-III**: AJ543781.1, AJ543789.1, AJ567871.1, AJ567872.1, AJ567877.1, AJ567880.1, CP017186.1; **C-IV**: AJ543784.1, AJ543806.1, AJ543807.1, AJ543867.1, AJ543877.1, AJ543889.1, AJ543893.1, CP017184.1; **C-V**: AJ417114.1, AJ862859.1, AJ862861.1, AJ862862.1, AJ862863.1, CP017279.1; **C-VI**: AJ543778.1, AJ543782.1, AJ567878.1, AJ866507.1, CP017180.1, CP017183.1; **C-VII**: AJ417108.1, AJ862866.1, AJ862867.1, AJ866491.1, CAI28773.1, MKEQ00000000.1; **C-VIII**: AJ543821.1, AJ543849.1, AJ543857.1, AJ543908.1, AJ567884.1, AJ567889.1, AJ567890.1, AJ567892.1, CAD66305, CAI28810.1, CP017179.1; **C-IX**: AJ543819.1, AJ543820.1, AJ543878.1, AJ543881.1, CAD66281.1, CAD66282.1, LT992502; **C-XI**: AJ417139.1, AJ417142.1, AJ543768.1, AJ543855.1, NC014121.1; **C-XII**: AJ417143.1, AJ543817.1, AJ543847.1, AJ862872.1, CP003678.1; **C-xiii**: AJ417128.1, AJ543837.1, AJ543870.1, AJ543872.1, CAD66299.1, CAD66332.1, CAD66334.1, CAD99100.1, LVUF00000000, FYBB00000000, SJOO00000000, AEXB00000000, QZCT01000000, FYBA00000000; **C-XIV**: ERS2281247, ERS2281248, LXPT00000000.1, LZEN00000000, NSIZ00000000.1, SJON00000000.1; **UD1**: JDWG00000000.1, JDWH00000000.1, JUZJ00000000.1, JUZQ00000000; **UD2**: BBUQ00000000.1, CP021851.1, JZYX00000000.1, NPNR00000000; **UD3**: AZXZ00000000.1, FCNO00000000.1, JACW00000000.1, JWAU00000000; **UD4**: CP043318.1, JVIL00000000.1, JZKT00000000.1, LEDN00000000; **UD5**: AP019007.1; and **UD6**: MTKD00000000, QZCS00000000.

Supplementary Table 1 Details of non-clinical samples: sampling date, location, type of environment, farm characteristics.

Supplementary Table 2 Details of strains: *hsp60* partial sequence and results for antibiotic resistance. Resistance profiles were obtained against 16 antibiotics. Intermediate or resistant results are labeled “R”; “s” corresponds to a susceptible phenotype.

Supplementary Table 3 Details of genetic content of sequenced *E. hormaechei* subsp. *xiangfangensis* (C-VI – clade A; *n* = 42) and *E. hormaechei* subsp. *steigerwaltii* (C-VIII – clade B; *n* = 86).

Supplementary Table 4 Cephalosporinase gene complex mutation. ^∗^Clone numbers refer to letters used in Figures 1, 2 and correspond to two strains from the same sample with different resistance profile against beta-lactam antibiotics: wild-type or cephalosporinase overproduction. Each strain is considered to be a clone that differs by < 45 single nucleotide polymorphisms (SNPs). na: not attributed. The abbreviation “N/S” after the gene names indicates the number of non-synonymous mutations (N) versus the number of synonymous mutations (S) in comparison to the reference data. Please note that only the different non-synonymous mutations between strains from the same sample have been conserved in the observation column.

## Abbreviations

3GC: third-generation cephalosporin
3GC-R: third-generation cephalosporin resistant
ECC: *Enterobacter cloacae* complex
ESBL: extended-spectrum beta-lactamase
ESKAPE: *Enterococcus faecium, Staphylococcus aureus, Klebsiella pneumoniae, Acinetobacter baumannii, Pseudomonas aeruginosa* and *Enterobacter* spp.
CoP: cephalosporinase overproduction
*hsp60*: heat-shock protein 60 gene-fragment
SNP: single nucleotide polymorphism
ST: sequence type
UD: undefined cluster
WGS: whole-genome sequencing
WT: wild-type.
