# Supplementary material for: Wide Distribution and Specific Resistance Pattern to Third-Generation Cephalosporins of Enterobacter cloacae Complex Members in Humans and in the Environment in Guadeloupe (French West Indies)
Source: Front Microbiol. 2021 Jun 25;12:628058. doi: 10.3389/fmicb.2021.628058 (PMC8268024; doi:10.3389/fmicb.2021.628058)
Supplement: Supplementary Figure 1 — Maximum likelihood phylogenetic tree based on the partial sequence hsp60 gene of E. cloacae complex (ECC) isolates recovered in Guadeloupe (n = 313). Maximum likelihood phylogenetic reconstructions were performed with RAxML software (100 bootstrap replicates), and the tree rooted with Klebsiella aerogenes hsp60 sequence (AB008141.1) was drawn with iTOL. All the sequences used as reference in the tree have a corresponding GenBank accession number, and only sequences extracted from whole-genome of ECC strains were labeled with current names. Each hsp60 cluster is identified by a specific color, and bootstrap values ≥ 60 are indicated. Hsp60 sequences from C-X, which correspond to Lelliottia nimipressuralis (formerly Enterobacter nimipressuralis) are inserted (AJ567887.1, AJ567900.1). For better readability, C-IV nodes are grouped. The hosts of each local strain are specified by a circle (human), a square (livestock), a star (fresh produce), a right-pointing triangle (water), or a left-pointing triangle (Anolis). Phenotypes are indicated by a white form for wild-type against beta-lactam antibiotics, and a black form for third-generation cephalosporin-resistant strains. The global distribution is provided in Table 3, and all hsp60 partial sequences are available in Supplementary Table 2. List of ECC partial hps60 sequences included in this figure (associated cluster: C or UD, and accession number): C-I: AJ417140.1, AJ567846.1, AJ567893.1, FXLQ00000000; C-II: AJ567849.1, AJ567862.1, AJ567886.1, AJ567888.1, AJ567899.1, CP017181.1, C-III: AJ543781.1, AJ543789.1, AJ567871.1, AJ567872.1, AJ567877.1, AJ567880.1, CP017186.1; C-IV: AJ543784.1, AJ543806.1, AJ543807.1, AJ543867.1, AJ543877.1, AJ543889.1, AJ543893.1, CP017184.1; C-V: AJ417114.1, AJ862859.1, AJ862861.1, AJ862862.1, AJ862863.1, CP017279.1; C-VI: AJ543778.1, AJ543782.1, AJ567878.1, AJ866507.1, CP017180.1, CP017183.1; C-VII: AJ417108.1, AJ862866.1, AJ862867.1, AJ866491.1, CAI28773.1, MKEQ00000000.1; C-VIII: AJ543821.1 [file Image_1.pdf]

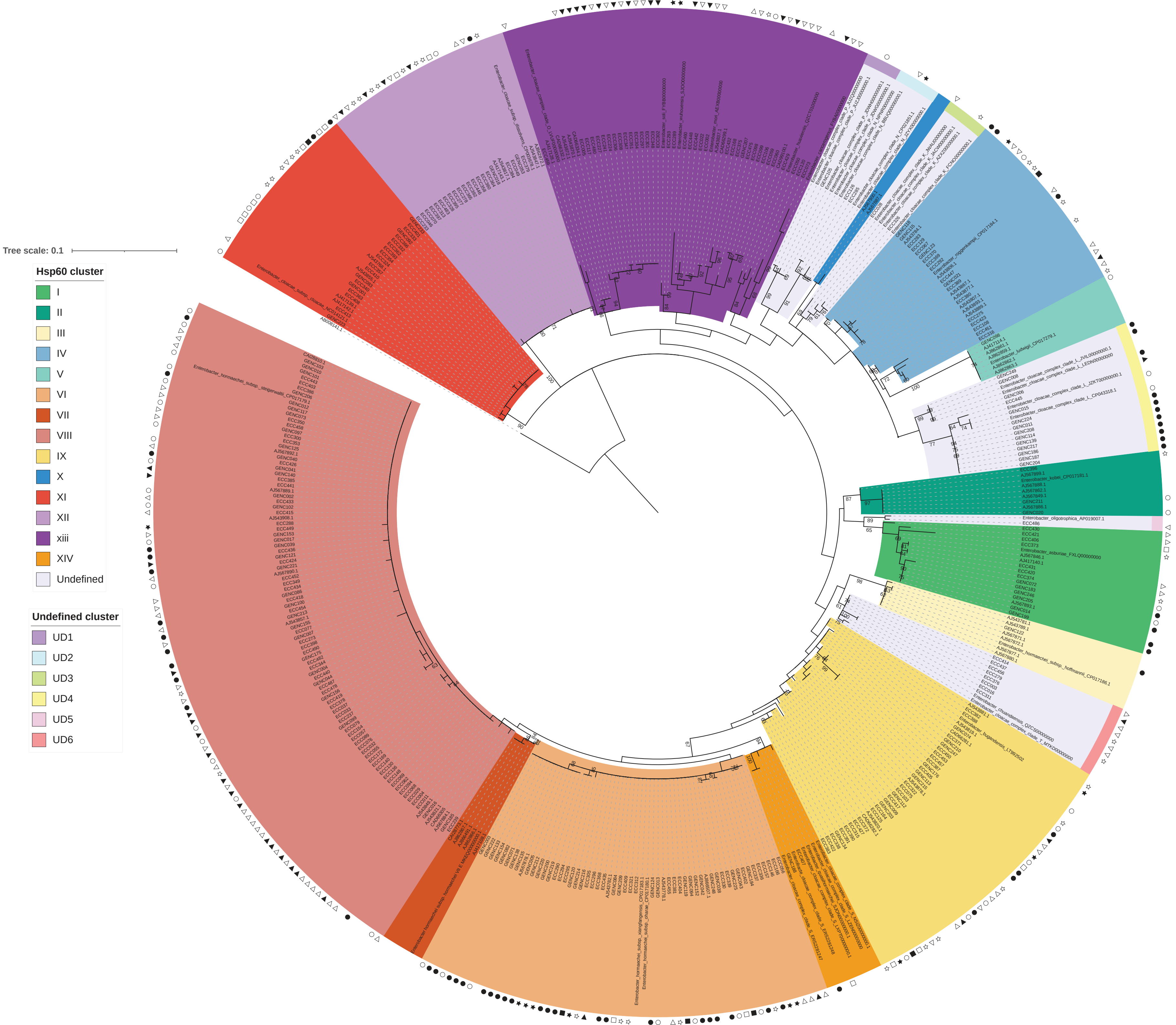

Tree scale: 0.1
Hsp60 cluster
I
II
III
IV
V
VI
VII
VIII
IX
X
XI
XII
xiii
XIV
Undefined
Undefined cluster
UD1
UD2
UD3
UD4
UD5
UD6

Supplementary Figure 1. Maximum likelihood phylogenetic tree based on the partial sequence *hsp60* gene of *E. cloacae* complex (ECC) isolates recovered in Guadeloupe (n=313).
Maximum likelihood phylogenetic reconstructions were performed with RAxML software (100 bootstrap replicates), and the tree rooted with *Klebsiella aerogenes hsp60* sequence (AB008141.1) was drawn with iTOL. All the sequences used as reference in the tree have a corresponding GenBank accession number, and only sequences extracted from whole-genome of ECC strains were labelled with current names. Each *hsp60* cluster is identified by a specific color, and bootstrap values ≥ 60 are indicated. *Hsp60* sequences from C-X, which correspond to *Lelliottia nimipressuralis* (formerly *Enterobacter nimipressuralis*) are inserted (AJ567887.1, AJ567900.1). For better readability, C-IV nodes are grouped. The hosts of each local strain are specified by a circle (human), a square (livestock), a star (fresh produce), a right-pointing triangle (water), or a left-pointing triangle (*Anolis*). Phenotypes are indicated by a white form for wild-type against beta-lactam antibiotics, and a black form for third-generation cephalosporin-resistant strains. The global distribution is provided in **Table 3**, and all *hsp60* partial sequences are available in **Supplementary Table 2**.

List of ECC partial *hps60* sequences included in this figure (associated cluster: C or UD, and accession number):

C-I: AJ417140.1, AJ567846.1, AJ567893.1, FXLQ00000000; **C-II:** AJ567849.1, AJ567862.1, AJ567886.1, AJ567888.1, AJ567899.1, CP017181.1, **C-III:** AJ543781.1, AJ543789.1, AJ567871.1, AJ567872.1, AJ567877.1, AJ567880.1, CP017186.1; **C-IV:** AJ543784.1, AJ543806.1, AJ543807.1, AJ543867.1, AJ543877.1, AJ543889.1, AJ543893.1, CP017184.1; **C-V:** AJ417114.1, AJ862859.1, AJ862861.1, AJ862862.1, AJ862863.1, CP017279.1; **C-VI:** AJ543778.1, AJ543782.1, AJ567878.1, AJ866507.1, CP017180.1, CP017183.1; **C-VII:** AJ417108.1, AJ862866.1, AJ862867.1, AJ866491.1, CAI28773.1, MKEQ00000000.1; **C-VIII:** AJ543821.1, AJ543849.1, AJ543857.1, AJ543908.1, AJ567884.1, AJ567889.1, AJ567890.1, AJ567892.1, CAD66305, CAI28810.1, CP017179.1; **C-IX:** AJ543819.1, AJ543820.1, AJ543878.1, AJ543881.1, CAD66281.1, CAD66282.1, LT992502; **C-XI:** AJ417139.1, AJ417142.1, AJ543768.1, AJ543855.1, NC014121.1; **C-XII:** AJ417143.1, AJ543817.1, AJ543847.1, AJ862872.1, CP003678.1; **C-xiii:** AJ417128.1, AJ543837.1, AJ543870.1, AJ543872.1, CAD66299.1, CAD66332.1, CAD66334.1, CAD99100.1, LVUF00000000, FYBB00000000, SJOO00000000, AEXB00000000, QZCT01000000, FYBA00000000; **C-XIV:** ERS2281247, ERS2281248, LXPT00000000.1, LZEN00000000, NSIZ00000000.1, SJON00000000.1; **UD1:** JDWG00000000.1, JDWH00000000.1, JUZJ00000000.1, JUZQ00000000; **UD2:** BBUQ00000000.1, CP021851.1, JZYX00000000.1, NPNR00000000; **UD3:** AZXZ00000000.1, FCNO00000000.1, JACW00000000.1, JWAU00000000; **UD4:** CP043318.1, JVIL00000000.1, JZKT00000000.1, LEDN00000000; **UD5:** AP019007.1; **UD6:** MTKD00000000, QZCS00000000.

Supplementary Figure 1 is detailed on the following images:

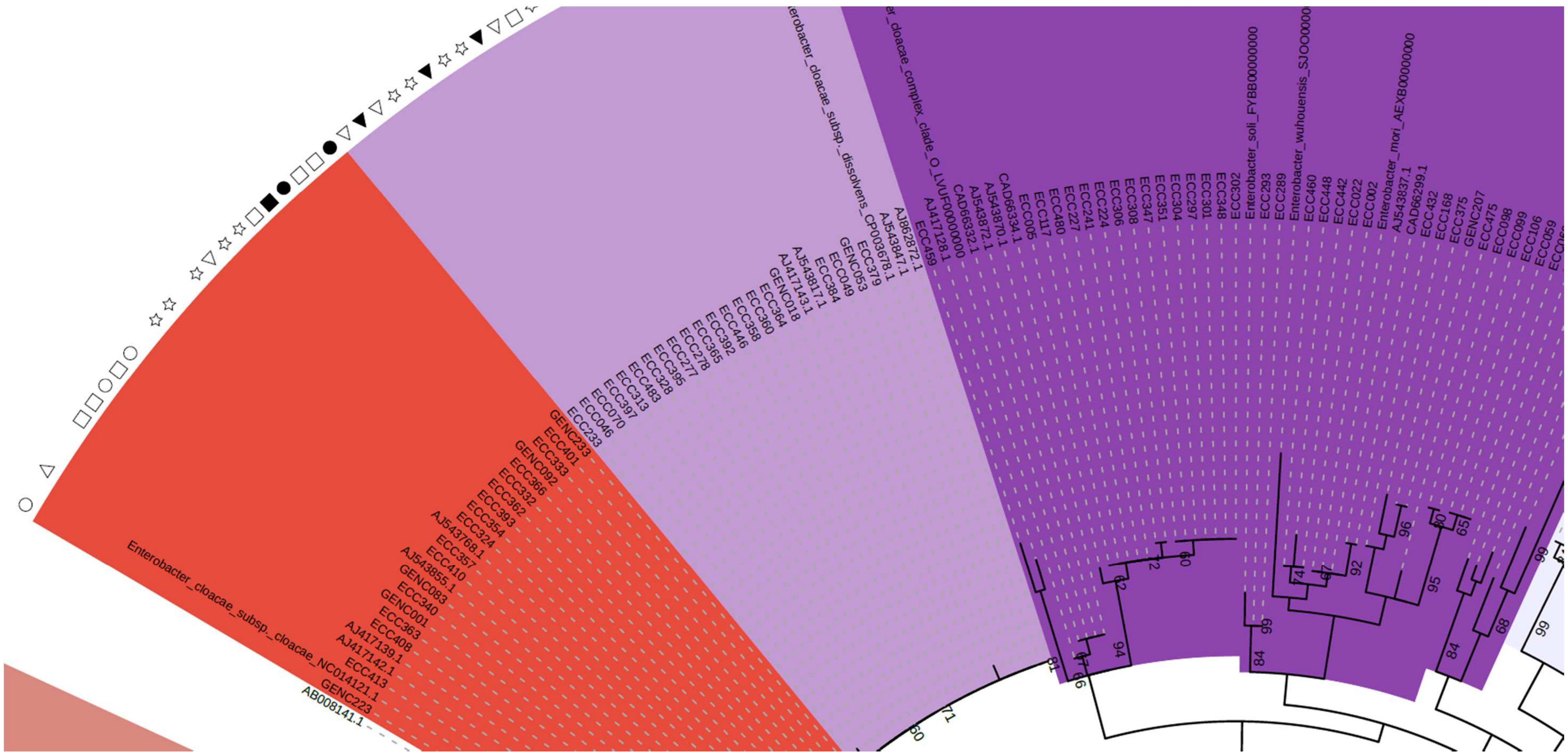

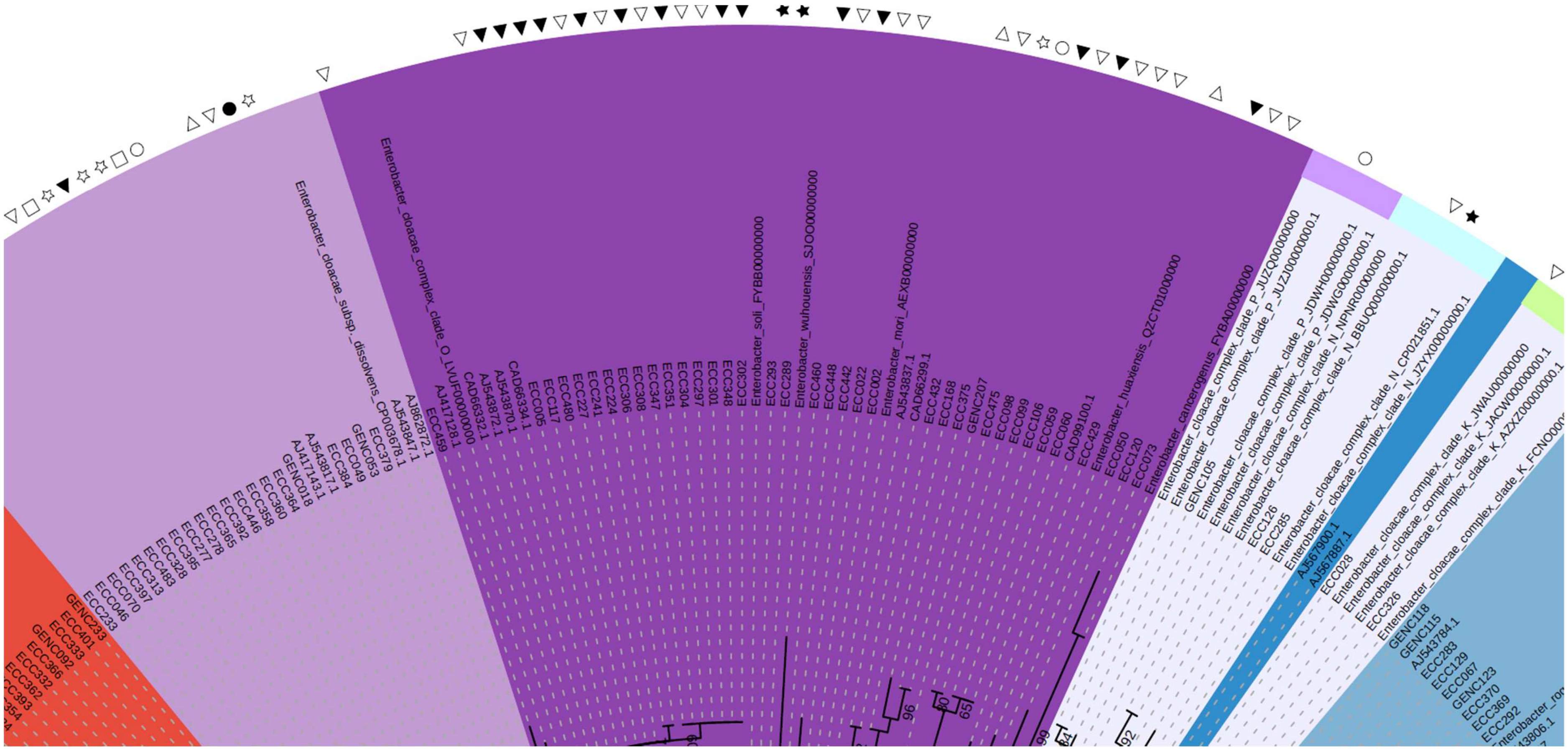
Enterobacter_cloacae_subsp._dissolvens_CP003678.1
AJ862872.1
AJ543847.1
ECC379
GENC053
ECC049
ECC384
AJ543817.1
AJ417143.1
GENC018
ECC364
ECC360
ECC358
ECC446
ECC392
ECC365
ECC278
ECC277
ECC395
ECC328
ECC483
ECC313
ECC397
ECC070
ECC046
ECC233
GENC233
ECC401
ECC333
GENC092
ECC366
ECC332
ECC362
Enterobacter_cloacae_complex_clade_O_LVUF00000000
ECC459
AJ417128.1
CAD66332.1
AJ543872.1
AJ543870.1
CAD66334.1
ECC005
ECC117
ECC480
ECC227
ECC241
ECC224
ECC306
ECC308
ECC347
ECC351
ECC304
ECC297
ECC301
ECC348
ECC302
Enterobacter_soli_FYBB00000000
ECC293
ECC289
Enterobacter_wuhouensis_SJOO00000000
ECC460
ECC448
ECC442
ECC022
ECC002
Enterobacter_mori_AEXB00000000
AJ543837.1
CAD66299.1
ECC432
ECC168
ECC375
GENC207
ECC475
ECC098
ECC099
ECC106
ECC059
ECC060
CAD99100.1
ECC429
Enterobacter_huaxiensis_QZCT01000000
ECC050
ECC120
ECC073
Enterobacter_cancerogenus_FYBA00000000
Enterobacter_cloacae_complex_clade_P_JUZQ00000000
Enterobacter_cloacae_complex_clade_P_JUZJ00000000.1
GENC105
Enterobacter_cloacae_complex_clade_P_JDWH00000000.1
Enterobacter_cloacae_complex_clade_P_JDWG00000000.1
Enterobacter_cloacae_complex_clade_N_NPNR00000000
Enterobacter_cloacae_complex_clade_N_BBUQ00000000.1
ECC126
ECC285
Enterobacter_cloacae_complex_clade_N_CP021851.1
Enterobacter_cloacae_complex_clade_N_JZYX00000000.1
AJ567900.1
AJ567887.1
ECC028
Enterobacter_cloacae_complex_clade_K_JWAU00000000
Enterobacter_cloacae_complex_clade_K_JACW00000000.1
Enterobacter_cloacae_complex_clade_K_AZXZ00000000.1
ECC326
GENC118
GENC115
AJ543784.1
ECC283
ECC129
ECC067
GENC123
ECC370
ECC369
ECC292
96
80
65
99
92

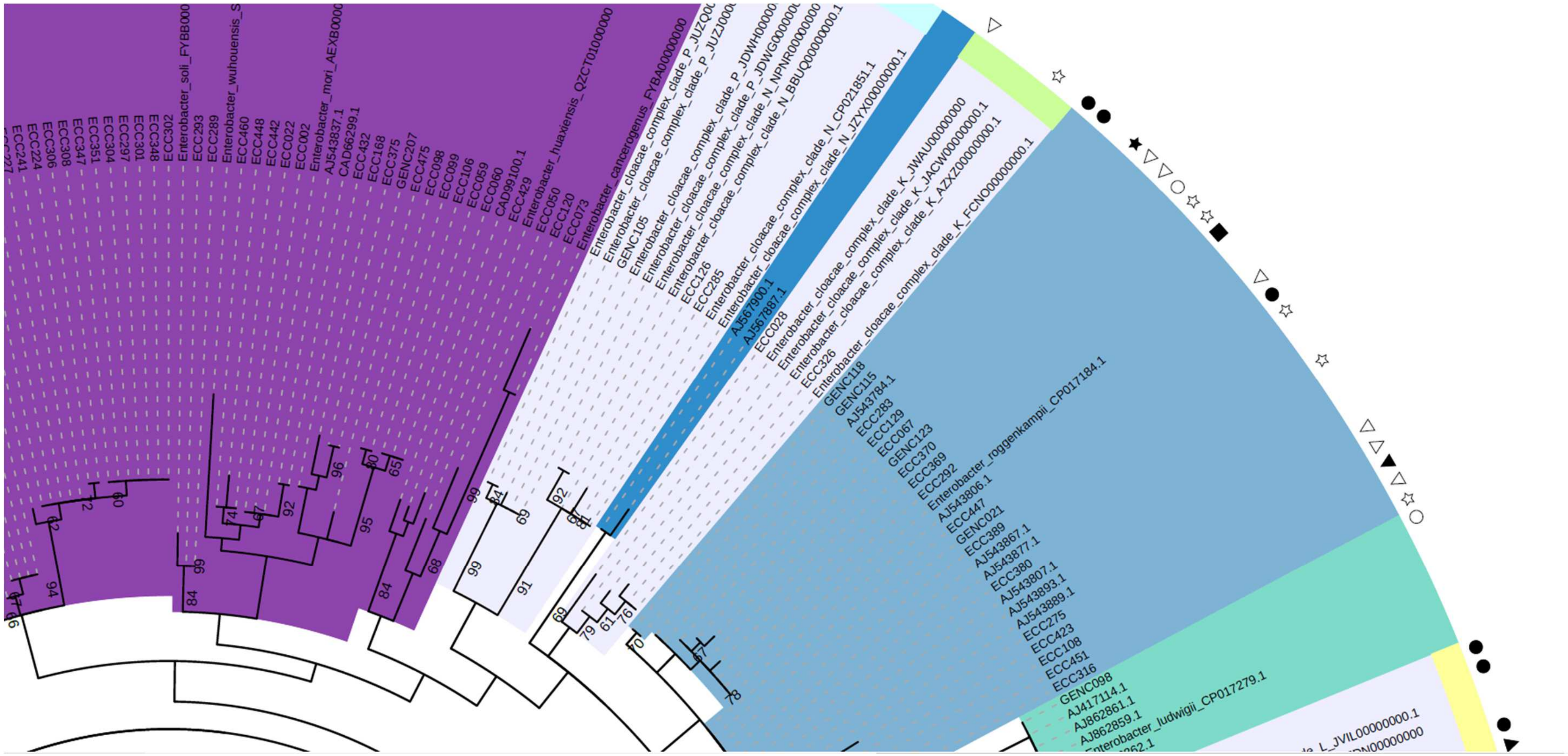
ECC241
ECC224
ECC306
ECC308
ECC347
ECC351
ECC304
ECC297
ECC301
ECC348
ECC302
ECC293
ECC289
ECC460
ECC448
ECC442
ECC022
ECC002
AJ543837.1
CAD66299.1
ECC432
ECC168
ECC375
GENC207
ECC475
ECC098
ECC099
ECC106
ECC059
ECC060
CAD99100.1
ECC429
Enterobacter_huaxiensis_QZCT01000000
ECC050
ECC120
ECC073
Enterobacter_cancerogenus_FYBA00000000
GENC105
ECC126
ECC285
Enterobacter_cloacae_complex_clade_N_CP021851.1
Enterobacter_cloacae_complex_clade_N_JZYX00000000.1
AJ567900.1
AJ567887.1
ECC028
ECC326
GENC118
GENC115
AJ543784.1
ECC283
ECC129
ECC067
GENC123
ECC370
ECC369
ECC292
Enterobacter_roggenkampii_CP017184.1
AJ543806.1
ECC447
GENC021
ECC389
AJ543867.1
AJ543877.1
ECC380
AJ543807.1
AJ543893.1
AJ543889.1
ECC275
ECC423
ECC108
ECC451
ECC316
GENC098
AJ417114.1
AJ862861.1
AJ862859.1
Enterobacter_ludwigii_CP017279.1

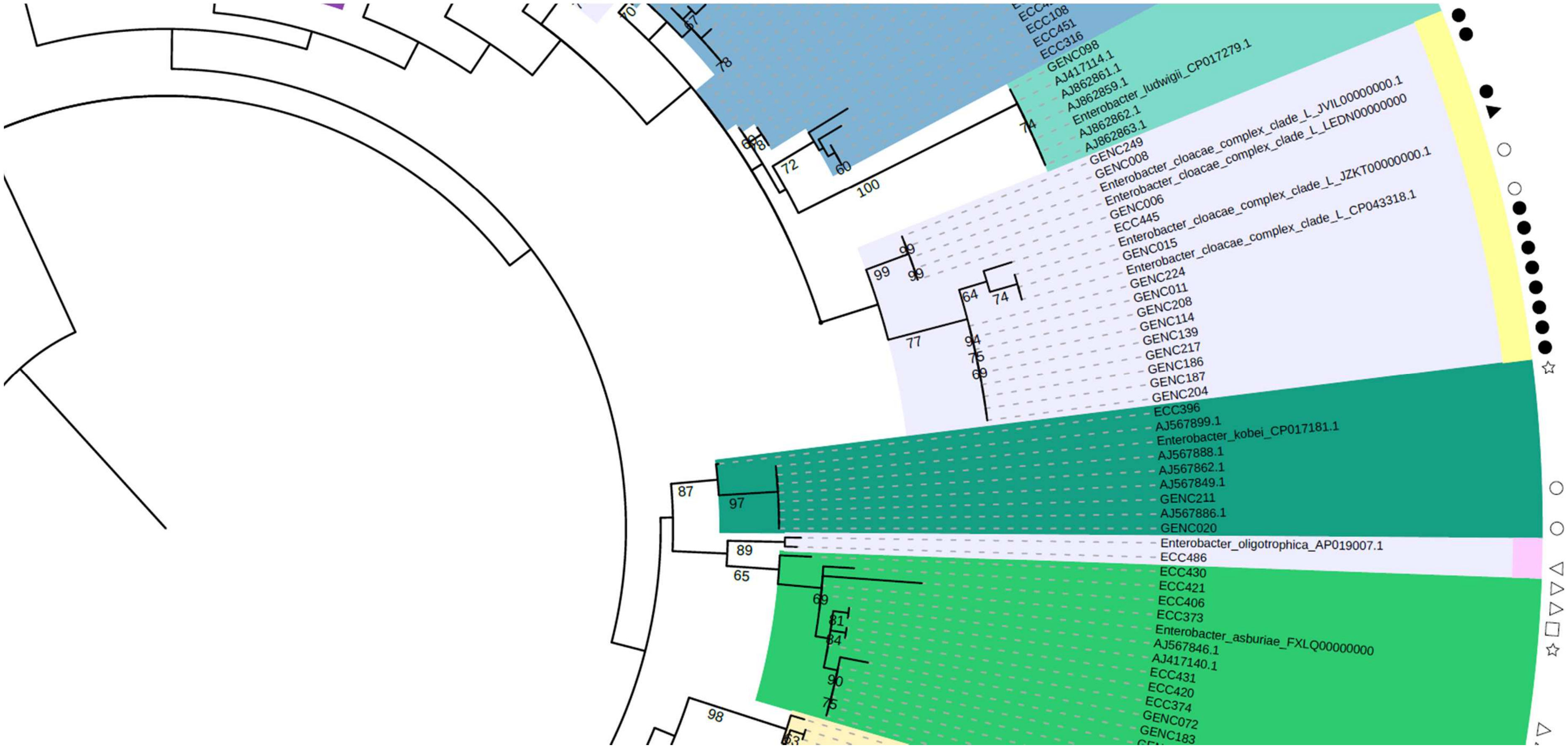
ECC108
ECC451
ECC316
GENC098
AJ417114.1
AJ862861.1
AJ862859.1
Enterobacter_ludwigii_CP017279.1
AJ862862.1
AJ862863.1
GENC249
GENC008
Enterobacter_cloacae_complex_clade_L_JVIL00000000.1
Enterobacter_cloacae_complex_clade_L_LEDN00000000
GENC006
ECC445
Enterobacter_cloacae_complex_clade_L_JZKT00000000.1
GENC015
Enterobacter_cloacae_complex_clade_L_CP043318.1
GENC224
GENC011
GENC208
GENC114
GENC139
GENC217
GENC186
GENC187
GENC204
ECC396
AJ567899.1
Enterobacter_kobei_CP017181.1
AJ567888.1
AJ567862.1
AJ567849.1
GENC211
AJ567886.1
GENC020
Enterobacter_oligotrophica_AP019007.1
ECC486
ECC430
ECC421
ECC406
ECC373
Enterobacter_asburiae_FXLQ00000000
AJ567846.1
AJ417140.1
ECC431
ECC420
ECC374
GENC072
GENC183

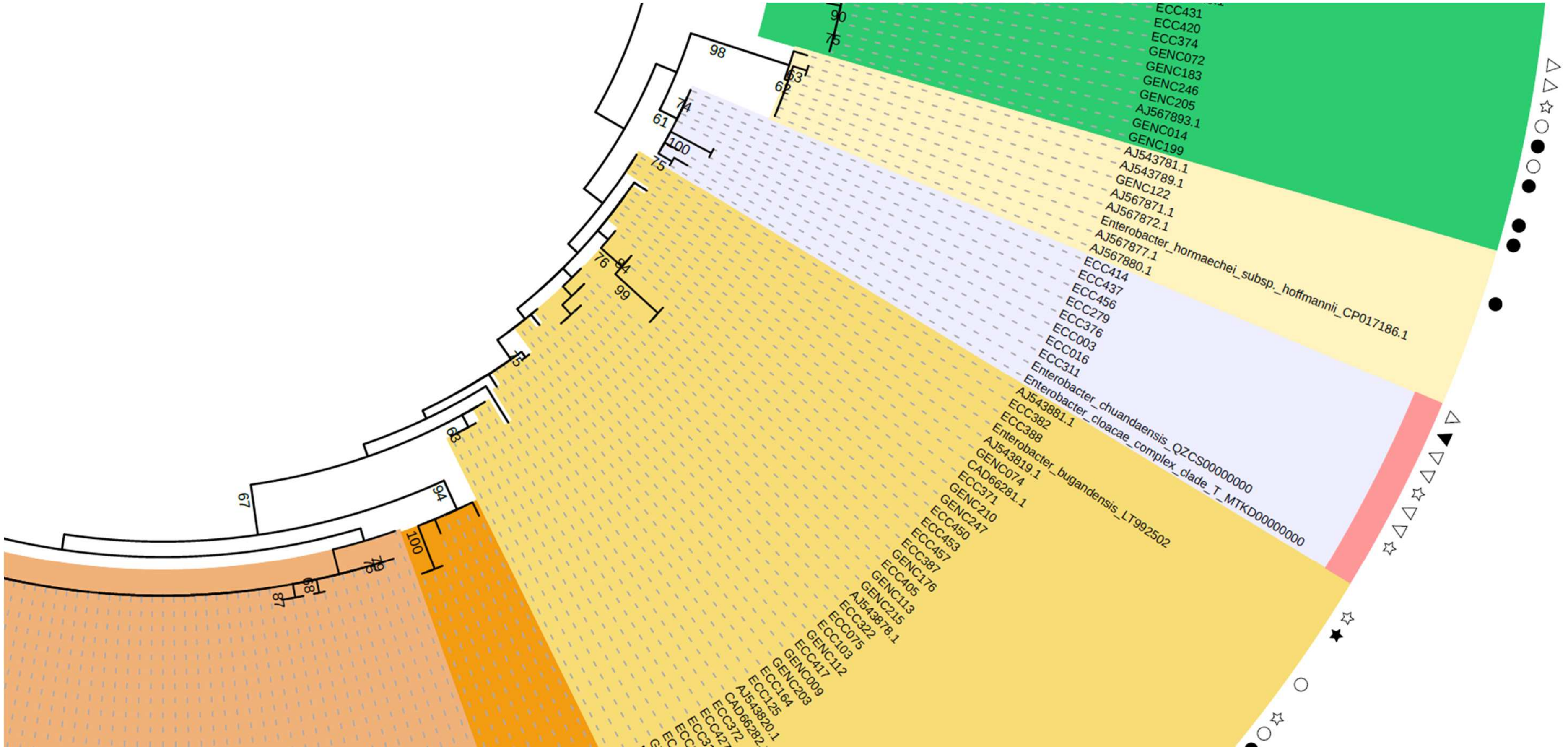
ECC431
ECC420
ECC374
GENC072
GENC183
GENC246
GENC205
AJ567893.1
GENC014
GENC199
AJ543781.1
AJ543789.1
GENC122
AJ567871.1
AJ567872.1
Enterobacter_hormaechei_subsp._hoffmannii_CP017186.1
AJ567877.1
AJ567880.1
ECC414
ECC437
ECC456
ECC279
ECC376
ECC003
ECC016
ECC311
Enterobacter_chuandaensis_QZCS00000000
Enterobacter_cloacae_complex_clade_T_MTKD00000000
AJ543881.1
ECC382
ECC388
Enterobacter_bugandensis_LT992502
AJ543819.1
GENC074
CAD66281.1
ECC371
GENC210
GENC247
ECC450
ECC453
ECC457
ECC387
GENC176
ECC405
GENC113
GENC215
AJ543878.1
ECC322
ECC075
ECC103
GENC112
ECC417
GENC009
GENC203
ECC164
ECC125
AJ543820.1
98
90
76
63
62
74
61
100
75
76
94
99
75
63
67
94
100
79
68
87

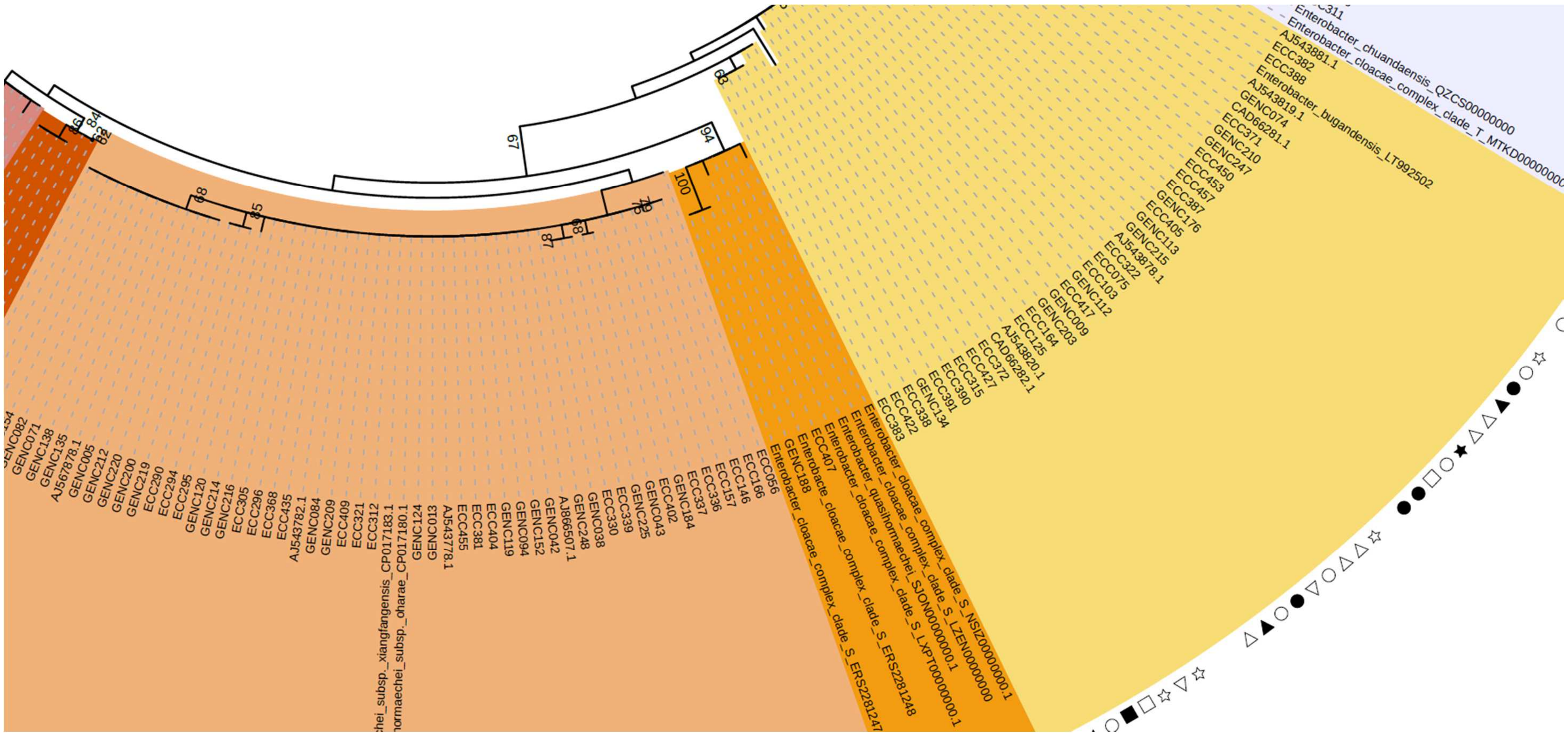
GENC082
GENC071
GENC138
GENC135
AJ567878.1
GENC005
GENC212
GENC220
GENC200
GENC219
ECC290
ECC294
ECC295
GENC120
GENC214
GENC216
ECC305
ECC296
ECC368
ECC435
AJ543782.1
GENC084
GENC209
ECC409
ECC321
ECC312
GENC124
GENC013
AJ543778.1
ECC455
ECC381
ECC404
GENC119
GENC094
GENC152
GENC042
AJ866507.1
GENC248
GENC038
ECC330
ECC339
GENC225
GENC043
ECC402
GENC184
ECC337
ECC336
ECC157
ECC146
ECC166
ECC056
Enterobacter_cloacae_complex_clade_S_ERS2281247
GENC188
Enterobacte_cloacae_complex_clade_S_ERS2281248
ECC407
Enterobacter_cloacae_complex_clade_S_LXPT00000000.1
Enterobacter_quasihormaechei_SJON00000000.1
Enterobacter_cloacae_complex_clade_S_LZEN00000000
Enterobacter_cloacae_complex_clade_S_NSIZ00000000.1
ECC383
ECC422
ECC338
GENC134
ECC391
ECC390
ECC315
ECC427
ECC372
CAD66282.1
AJ543820.1
ECC125
ECC164
GENC203
GENC009
ECC417
GENC112
ECC103
ECC075
ECC322
AJ543878.1
GENC215
GENC113
ECC405
GENC176
ECC387
ECC457
ECC453
ECC450
GENC247
GENC210
ECC371
CAD66281.1
GENC074
AJ543819.1
Enterobacter_bugandensis_LT992502
ECC388
ECC382
AJ543881.1
Enterobacter_chuandaensis_QZCS00000000
100
94
67
63

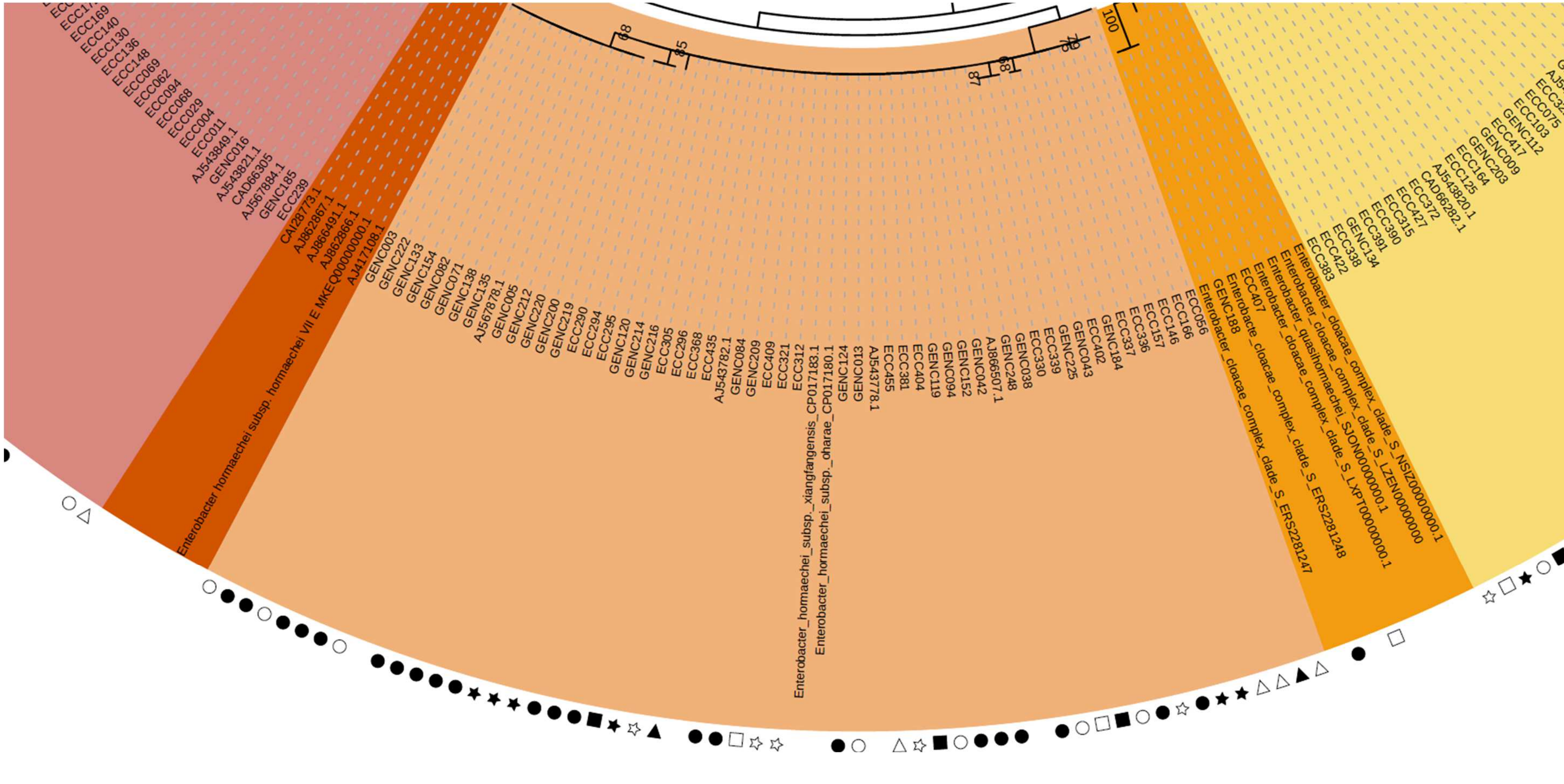
ECC169
ECC140
ECC130
ECC136
ECC148
ECC069
ECC062
ECC094
ECC068
ECC029
ECC004
ECC011
AJ543849.1
GENC016
AJ543821.1
CAD66305
AJ567884.1
GENC185
ECC239
CAI28773.1
AJ862867.1
AJ866491.1
AJ862866.1
Enterobacter hormaechei subsp. hormaechei VII E MKEQ00000000.1
AJ417108.1
GENC003
GENC222
GENC133
GENC154
GENC082
GENC071
GENC138
GENC135
AJ567878.1
GENC005
GENC212
GENC220
GENC200
GENC219
ECC290
ECC294
ECC295
GENC120
GENC214
GENC216
ECC305
ECC296
ECC368
ECC435
AJ543782.1
GENC084
GENC209
ECC409
ECC321
ECC312
Enterobacter_hormaechei_subsp._xiangfangensis_CP017183.1
Enterobacter_hormaechei_subsp._oharae_CP017180.1
GENC124
GENC013
AJ543778.1
ECC455
ECC381
ECC404
GENC119
GENC094
GENC152
GENC042
AJ866507.1
GENC248
GENC038
ECC330
ECC339
GENC225
GENC043
ECC402
GENC184
ECC337
ECC336
ECC157
ECC146
ECC166
ECC056
Enterobacter_cloacae_complex_clade_S_ERS2281247
GENC188
Enterobacte_cloacae_complex_clade_S_ERS2281248
ECC407
Enterobacter_cloacae_complex_clade_S_LXPT00000000.1
Enterobacter_quasihormaechei_SJON00000000.1
Enterobacter_cloacae_complex_clade_S_LZEN00000000
Enterobacter_cloacae_complex_clade_S_NSIZ00000000.1
ECC383
ECC422
ECC338
GENC134
ECC391
ECC390
ECC315
ECC427
ECC372
CAD66282.1
AJ543820.1
ECC125
ECC164
GENC203
GENC009
ECC417
GENC112
ECC103
ECC075
ECC322
68
85
87
100

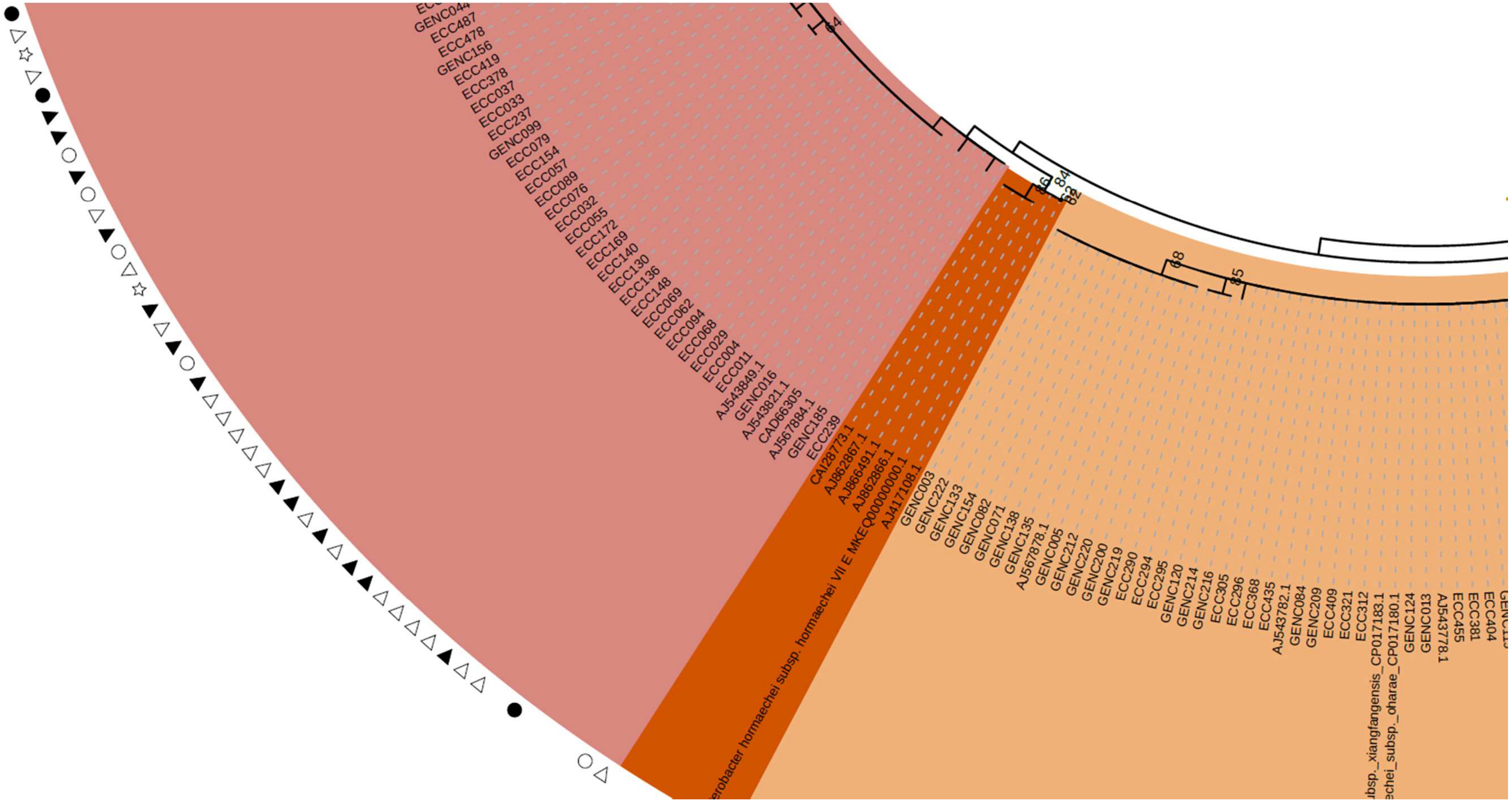

GENC044
ECC487
ECC478
GENC156
ECC419
ECC378
ECC037
ECC033
ECC237
GENC099
ECC079
ECC154
ECC057
ECC089
ECC076
ECC032
ECC055
ECC172
ECC169
ECC140
ECC130
ECC136
ECC148
ECC069
ECC062
ECC094
ECC068
ECC029
ECC004
ECC011
AJ543849.1
GENC016
AJ543821.1
CAD66305
AJ567884.1
GENC185
ECC239
CAI28773.1
AJ862867.1
AJ866491.1
AJ862866.1
MKEQ00000000.1
AJ417108.1
GENC003
GENC222
GENC133
GENC154
GENC082
GENC071
GENC138
GENC135
AJ567878.1
GENC005
GENC212
GENC220
GENC200
GENC219
ECC290
ECC294
ECC295
GENC120
GENC214
GENC216
ECC305
ECC296
ECC368
ECC435
AJ543782.1
GENC084
GENC209
ECC409
ECC321
ECC312
GENC124
GENC013
AJ543778.1
ECC455
ECC381
ECC404
64
86
84
62
68
85

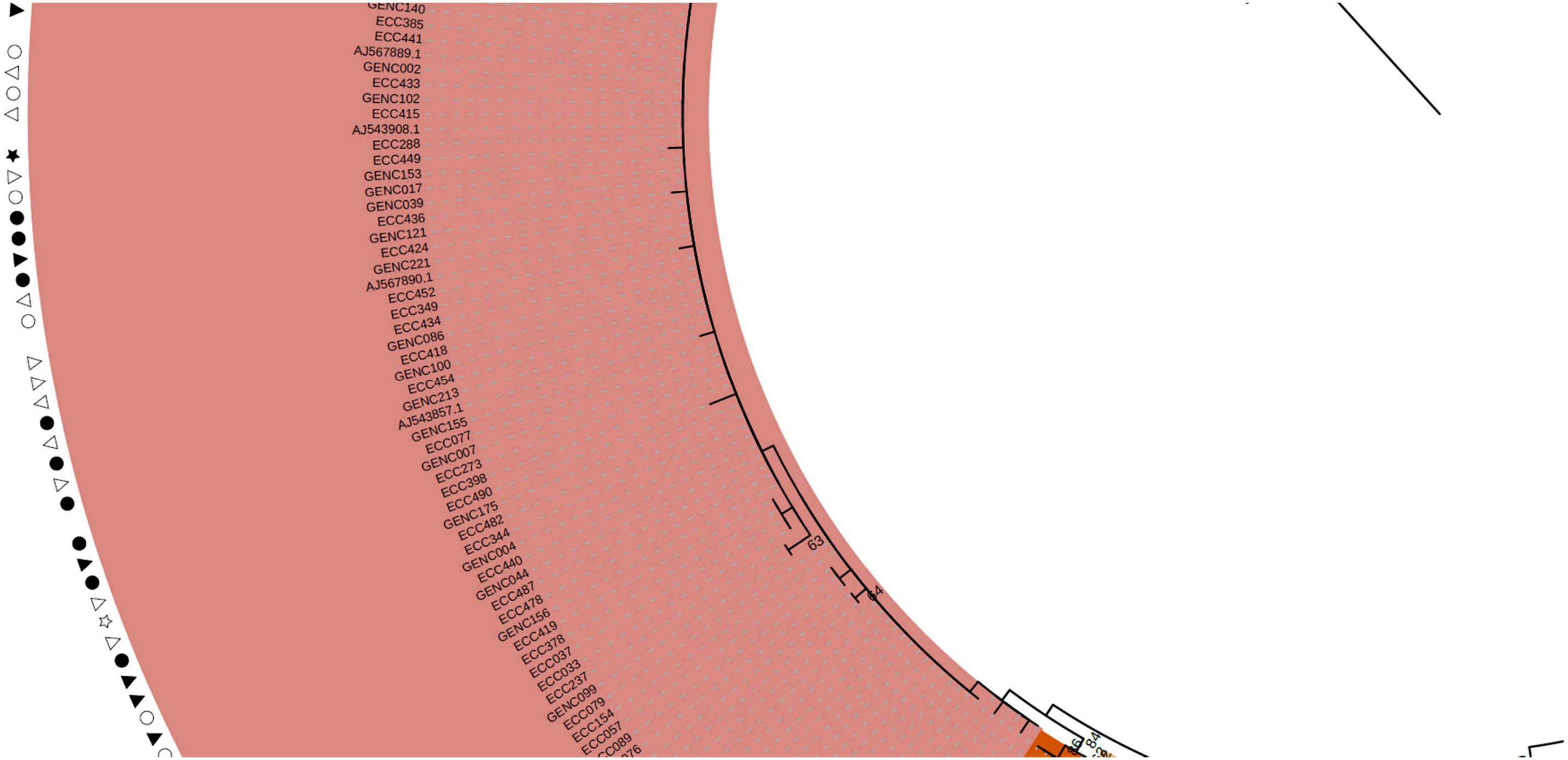
GENC140
ECC385
ECC441
AJ567889.1
GENC002
ECC433
GENC102
ECC415
AJ543908.1
ECC288
ECC449
GENC153
GENC017
GENC039
ECC436
GENC121
ECC424
GENC221
AJ567890.1
ECC452
ECC349
ECC434
GENC086
ECC418
GENC100
ECC454
GENC213
AJ543857.1
GENC155
ECC077
GENC007
ECC273
ECC398
ECC490
GENC175
ECC482
ECC344
GENC004
ECC440
GENC044
ECC487
ECC478
GENC156
ECC419
ECC378
ECC037
ECC033
ECC237
GENC099
ECC079
ECC154
ECC057
63

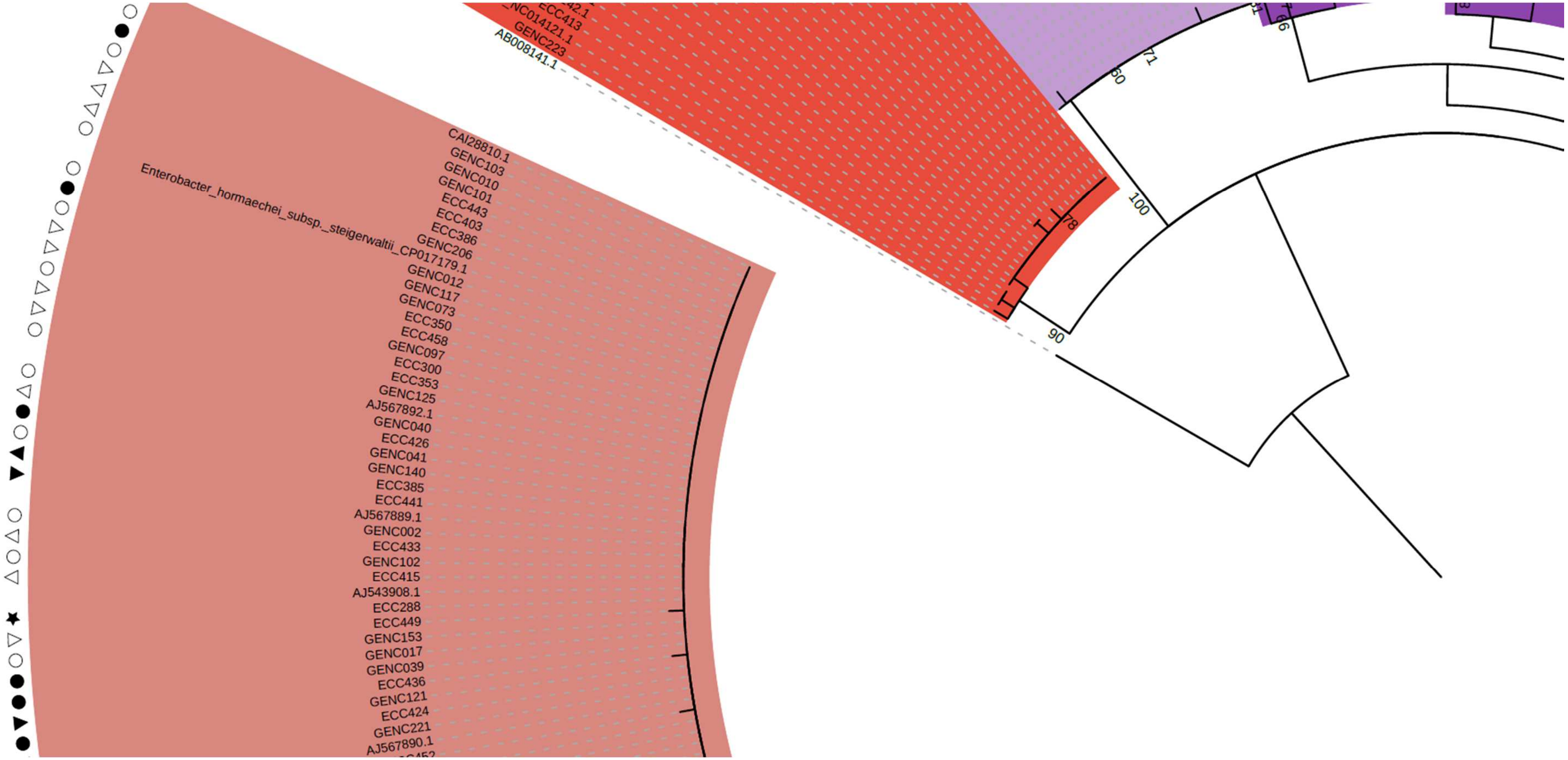

ECC413
GENC223
AB008141.1
CAI28810.1
GENC103
GENC010
GENC101
ECC443
ECC403
ECC386
GENC206
Enterobacter_hormaechei_subsp._steigerwaltii_CP017179.1
GENC012
GENC117
GENC073
ECC350
ECC458
GENC097
ECC300
ECC353
GENC125
AJ567892.1
GENC040
ECC426
GENC041
GENC140
ECC385
ECC441
AJ567889.1
GENC002
ECC433
GENC102
ECC415
AJ543908.1
ECC288
ECC449
GENC153
GENC017
GENC039
ECC436
GENC121
ECC424
GENC221
AJ567890.1
100
90
78
60
71
66
